# Vibrational spectroscopy, structure and bonding in the family of aluminium-doped niobium clusters, AlNb_*n*_^+^, *n* = 5–9

**DOI:** 10.1039/d6cp01125j

**Published:** 2026-07-03

**Authors:** Roshan Singh, Piero Ferrari, Deepak Pradeep, Joost M. Bakker, André Fielicke, Ewald Janssens, Peter Lievens, John E. McGrady

**Affiliations:** a Department of Chemistry, University of Oxford South Parks Road Oxford OX1 3QR U.K john.mcgrady@chem.ox.ac.uk; b HFML-FELIX, Nijmegen 6525 ED The Netherlands piero.ferrariramirez@ru.nl; c Fritz-Haber-Institut der Max-Planck-Gesellschaft Faradayweg 4-6 14195 Berlin Germany; d Quantum Solid-State Physics, Department of Physics and Astronomy, KU Leuven Celestijnenlaan 200 D B-3001 Leuven Belgium ewald.janssens@kuleuven.be

## Abstract

Infrared multiple-photon dissociation (IR-MPD) spectroscopy is used to investigate the vibrational spectra of a series of Ar-tagged Nb/Al clusters, AlNb_*n*_^+^, *n* = 5–9. In the smaller cluster size regime (*n* = 5–7), the structures evolve through capping of the faces of an AlNb_5_^+^ octahedral core. The core octahedron is in all cases axially compressed, with a short Nb–Nb distance between two mutually *trans* vertices, and the infrared spectra can be assigned in terms of rigid translations of this Nb_2_ unit relative to the remainder of the cluster. The larger clusters, AlNb_8_^+^ and AlNb_9_^+^, are derived from the corresponding Nb_8_^+^ and Nb_9_^+^ clusters by capping of a rhombic face, and this structural feature correlates with the emergence of vibrational transitions above 300 cm^−1^.

## Abstract 2

Infrared multiple-photon dissociation (IR-MPD) spectroscopy is used to investigate the vibrational spectra of a series of Ar-tagged Nb/Al clusters, AlNb_*n*_^+^, *n* = 5–9.

## Graphical abstract

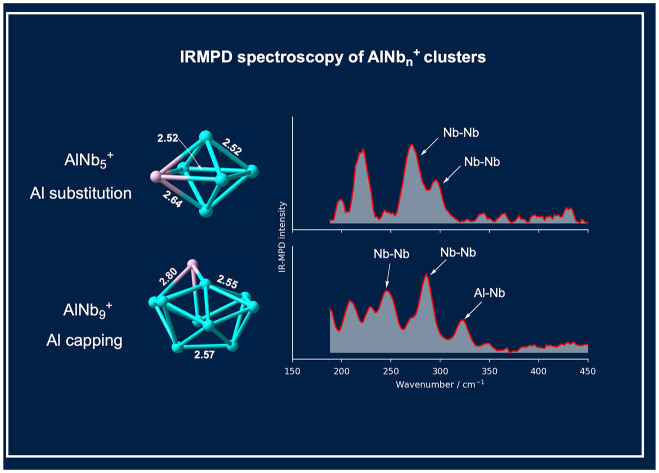

## Introduction

Hybrid materials containing both main-group and transition metals are of interest in a number of contexts, from materials science through metallurgy to catalysis.^1–3^ In studies of this class of material, the over-arching question is always the extent to which one element influences the other, and whether the composite material has properties that are more than the sum of the component parts. The focus of this paper is on mixed Nb/Al systems, and alloys of these two metals have a lengthy history, starting with the identification of Nb_3_Al as one of the earliest superconducting materials.^4^ The Nb/Al phase diagram is, however, complex,^5^ and different binary compositions including Nb_2_Al and NbAl_3_ are stable under different sets of conditions. At the opposite end of the length scale, small clusters, typically with fewer than 100 atoms, have offered valuable insights into structure, bonding and reactivity, and have also served as models for bulk materials. Some of the earliest work on mixed Al/Nb clusters came from Nonose *et al.*, who used time-of-flight mass spectrometry to explore the reactivity of neutral clusters with H_2_.^6^ Comparison to the all-Nb analogues showed that the addition of Al serves to reduce reactivity for small clusters (up to 7 Nb atoms) but precisely the opposite effect was observed in the larger members of the series. The authors argued that the addition of Al to the smaller clusters resulted in the blocking of active sites, but in the larger analogues it disrupted a more uniform surface, giving a locus for reactivity. The links between geometric structure and reactivity with small molecules (H_2_, CO) across a wider range of doped clusters were the subject of a 2018 review article by Ferrari *et al.*^7^

A broad range of spectroscopic tools have been applied to both pure Nb^8^ and mixed Nb/Al clusters, notably the photoelectron spectroscopy reported by Pramann *et al.*^9^ The authors showed that the impact of Al dopants on the ionisation energy is significant for small clusters (fewer than 9 Nb atoms) but much less for larger clusters, consistent with Nonose's earlier finding of a discontinuity in H_2_ absorption in the same size domain. At the opposite end of the Al/Nb composition scale, Yan *et al.* have very recently reported the photoelectron spectroscopy of the Al-rich family, NbAl_*n*_^0/−^, where the growth pattern can be understood in terms of capping of an Al_3_Nb core.^10^ Density functional theory has developed into an indispensable complement to gas-phase spectroscopy, and numerous reports on AlNb_*n*_^+/0/−1^ clusters have also appeared in the literature. Two 2012 papers by Wang and co-workers set out the structural landscape for neutral and anionic clusters with *n* = 2–10, where endohedral encapsulation of Al proves to be unfavourable, even in the largest examples.^11,12^ This resistance of small Nb clusters to encapsulation represents a striking contrast with early first-row transition metal analogues, where metal–metal bonding is somewhat weaker.^13–16^ More recently, Pansini and co-workers have explored the structural landscape for more Al-rich clusters, Al_*m*_Nb_*n*_, *m* = 0–5 and *n* = 4–10, where the most stable structures have the Al atoms widely dispersed over a Nb_*n*_ core. The per-atom binding energy in these clusters increases with size of the cluster, approaching a plateau around 4–5 eV for *m* + *n* = 10, and decreases as the Al content increases.^17^ Clearly, the position of the Al dopant on/in the cluster depends on a rather subtle balance between the cohesive energy of Nb and the strength of the heterometallic Al–Nb bond. A broader survey of transition metal encapsulation across a series of TMAl_*n*_^+^, *n* = 5–35, indicates that encapsulation of the transition metal atom occurs only when the number of Al atoms exceeds 16 (TM = V, Cr) or 19–21 (TM = Ti).^18^

In this paper, we report the infrared multiphoton-dissociation (IR-MPD) spectra of a family of Ar-tagged cationic Nb/Al clusters, AlNb_*n*_^+^, *n* = 5–9, and also of the corresponding all-Nb family, Nb_*n*_^+^, *n* = 6–10 which provides an important point of reference for the mixed-metal species. The Nb clusters have been the subject of a number of previous studies, most notably in a series of IR-MPD experiments reported by Fielicke and co-workers which covered both neutral and cationic clusters Nb_*n*_^0/+^ for *n* up to 9.^19,20^ These previous studies covered a wider frequency range than is used here but, where our new experiments do overlap with previously published work, the spectra are very similar (see Fig. S2). Nguyen and co-workers have authored a number of computational papers interpreting the IR-MPD spectra of the all-Nb clusters,^21–24^ including predictions for larger members Nb_10_^0/+^, Nb_11_^0/+^ and Nb_12_^0/+^ which, in turn, prompted Fielicke and co-workers to publish further experimental data that expanded the size range to Nb_12_.^25^ Here, we complement the IR-MPD spectroscopy on mixed Al/Nb clusters with a detailed survey of the potential energy surfaces of these clusters using DFT. Our computational work starts from the same point as many others, in that we seek first to identify the most plausible (*i.e.* low-energy) isomers that are consistent with the spectroscopy. Once a match has been established, we perform a careful analysis of the fundamental vibrational modes and show that the spectra of the smallest clusters AlNb_5_^+^, AlNb_6_^+^ and AlNb_7_^+^ can be interpreted in terms of rigid translations of an Nb_2_ unit. This structural motif is apparent in many of the previous DFT studies of Nb and Al/Nb clusters^11,12,17,21–24,26–32^ but its origins and significance have not previously been commented upon. For the larger clusters, a different growth pattern emerges: the Al atom is exohedrally bound to a rhombic face of a Nb_*n*_ cluster, the structure of which resembles closely the Al-free analogue with the same number of Nb atoms. The emergence of vibrational modes above 300 cm^−1^ appear to be diagnostic of this structural transition.

## Methodology

### Experimental techniques

Experiments are performed in a molecular beam set up coupled to the light of the free electron laser FELICE, part of the HFML-FELIX infrastructure in Nijmegen (the Netherlands). A distribution of pure Nb_*n*+1_^+^ and AlNb_*n*_^+^ clusters is produced in a dual-target dual-laser source by pulse ablation of Nb and Al plate targets. Cluster-Ar complexes are formed by condensation of the vaporized material in a short pulse of He gas containing roughly 2% of Ar, while the source is cooled down to 200 K by a continuous flow of liquid N_2_. After expansion through a conical nozzle, the molecular beam is collimated before perpendicular extraction into a reflectron time-of-mass spectrometer. A representative mass spectrum is shown in Fig. S1. Infrared (IR) spectra are recorded by merging the molecular beam with the light of FELICE in the free electron cavity, thus providing high power and a large interaction volume. Resonant absorption of IR light heats the cluster–Ar complexes through internal vibrational redistribution (IVR), which results in dissociation of the complex *via* its lowest dissociation channel, in this case Ar emission. The measured spectra therefore correspond to the cluster–Ar complex rather than the isolated cluster. Infrared multiple photon dissociation (IR-MPD) spectra are constructed by comparing mass spectra with and without the influence of FELICE, by running FELICE at half the repetition rate of the molecular beam experiment. The wavenumber is varied from 200 to 600 cm^−1^ in steps of 2 cm^−1^. The resonant absorption of IR light is then reflected in desorption of the Ar messenger atom leading to wavenumber-dependent depletion spectra, which are converted into an IR yield by:
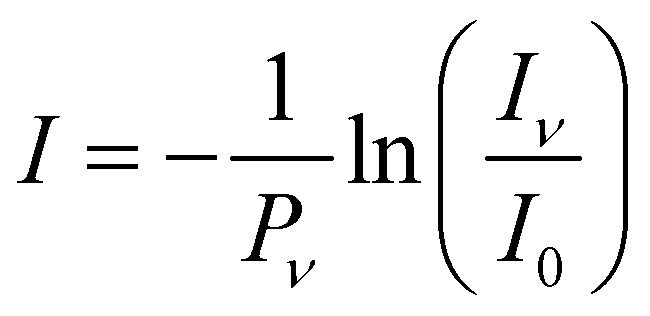
with *P*_*ν*_ is the wavelength dependent laser energy, and *I*_*ν*_ and *I*_0_ the intensity of AlNb_*n*_^+^·Ar cluster in mass spectra with and without laser excitation, respectively.

### Computational techniques

The initial geometries for the structures of the pure niobium clusters were taken from previous literature reports of Nb clusters before being optimised with the PBE functional.^17,21,22,24,33^ This functional has been used extensively in computational cluster chemistry, including in our own previous work predicting the ground states of Re/Si and Mn/Si clusters.^34,35^ The initial geometries for the aluminium doped clusters, AlNb_*n*_^+^, were then generated using two distinct strategies. In the first, we performed a systematic substitution of an Al atom in every symmetry-distinct Nb position of the pure Nb cluster with the same number of vertices *i.e.* Nb_*n*+1_^+^. In the second, we took the optimised structure of the all-Nb cluster with the same number of Nb atoms (*i.e.* Nb_*n*_^+^) and systematically placed an Al atom in a capping position above all symmetry-distinct faces, edges and vertices. In this way, we hope to sample the potential energy surface in an unbiased fashion. All optimisations were performed using the Amsterdam Density Functional (ADF) package, version 2021.104^36^ and a Slater-type triple-zeta basis set with two polarisation functions (TZ2P).^37^ The density was fitted using the QZ4P set of fit functions and a good quality Becke grid was used throughout for the numerical integration. Harmonic vibrational frequencies were computed at the equilibrium geometries using the same combination of functional and basis set, with each resonance broadened into a Lorentzian line shape of 5 cm^−1^ full width at half maximum to generate the plots.^35^ In all computed spectra, the frequencies are unscaled.

In previous work on vanadium and niobium clusters, Fielicke *et al.* have shown that the IRMPD spectra are not significantly perturbed by the presence of a rare gas atom^38^ and similar observations have been made for Rh_5_Ta(NO)^+^.^39^ and also for metallo-silicon clusters.^40^ Conversely, binding of an Ar atom to [Pt_2_O_3_]^+^ results in an energetic reordering of isomers.^41^ In our previous study of ReSi_*n*_^+^ clusters, the inclusion of the Xe tag was found to influence the intensity of the specific mode corresponding to the dissociation of the Re atoms from the Si_*n*_ unit.^34^ We have therefore also investigated the influence of Ar tag on the spectra of AlNb_5–9_^+^ – see SI, Fig. S4. In all cases the Ar atom binds to the apical Nb atom in the axially compressed Nb_2_ unit present in all the clusters AlNb_5–9_^+^, but the influence of the tag on computed frequencies is negligible (less than 5 cm^−1^). However, there is a minor increase in the intensity of some of the peaks in the 225 cm^−1^ region.

## Results and discussions

### Overview of the IR-MPD spectra of AlNb_5–9_^+^ and comparison to Nb_6–10_^+^

**Fig. 1:**
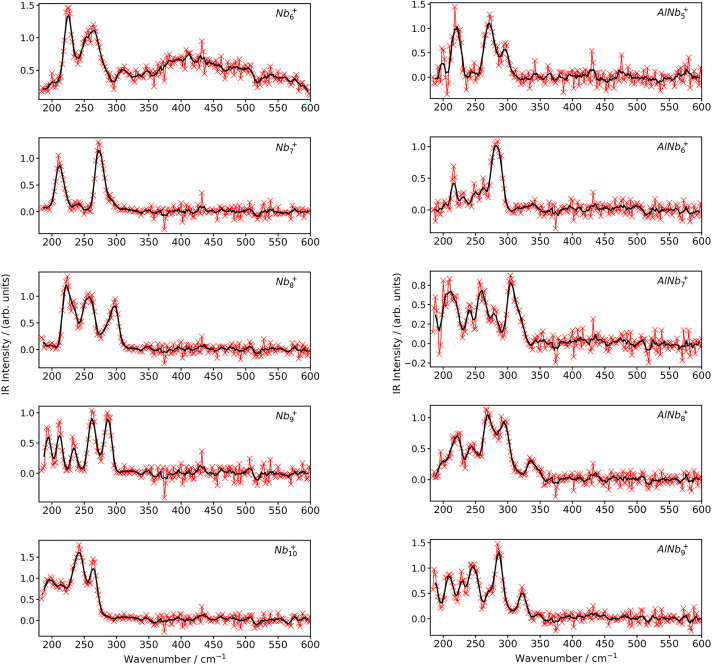
Comparison of the IR-MPD spectra of AlNb_*n*_^+^·Ar and Nb_*n*+1_^+^·Ar (*n* = 5–9) complexes. The black line is the 5 point average.

The experimental IR-MPD spectra for the Al-doped clusters, AlNb_*n*_^+^, *n* = 5–9, measured at HFML-FELIX, are collected in Fig. 1. The corresponding spectra for the all-Nb clusters of the same nuclearity, also measured at HFML-FELIX, are shown for comparison in the right hand panel. For the Nb_*n*_^+^ clusters, the spectra are largely featureless in the high-frequency region between 350 and 600 cm^−1^, consistent with our computational work (*vide infra*) which confirms that there are no fundamental vibrational modes significantly above 350 cm^−1^. The only exception is the Nb_6_^+^ cluster, where a broad band centred on 420 cm^−1^ is present: we discuss the origin of this band later in the manuscript. The spectra of Nb_*n*_^+^ reported here are very similar to those described by Fielicke *et al.*^19^ in a previous study: the two sets of spectra are compared in the SI (Fig. S2) and the very minor differences reflect subtle changes in the setup in the FELIX and FELICE experiments. Further detail is given in the SI. The spectra of the Al-doped clusters are richer than those of the corresponding all-Nb clusters with the same number of atoms, reflecting their generally lower symmetry, and they appear to fall into two quite distinct categories. For the lighter clusters, AlNb_5_^+^ and AlNb_6_^+^, the spectra in the 200–300 cm^−1^ window are strikingly similar to the all-Nb analogues of the same nuclearity, and there are no significant features above 300 cm^−1^. For the larger clusters, however, the similarities between the doped and undoped clusters are less obvious, and bands of significant intensity above 300 cm^−1^ appear in AlNb_7_^+^, AlNb_8_^+^ and AlNb_9_^+^. In the region between 200 and 300 cm^−1^, it is possible to discern two and sometimes three relatively intense features, with a number of additional less intense bands. In the following sections we make pairwise comparisons between clusters of the same nuclearity, Nb_*n*+1_^+^ with AlNb_*n*_^+^, assign and interpret the spectra, and then build a model to explain the evolution of structure and spectra across the series.

### AlNb_5_^+^ and comparison with Nb_6_^+^

**Fig. 2:**
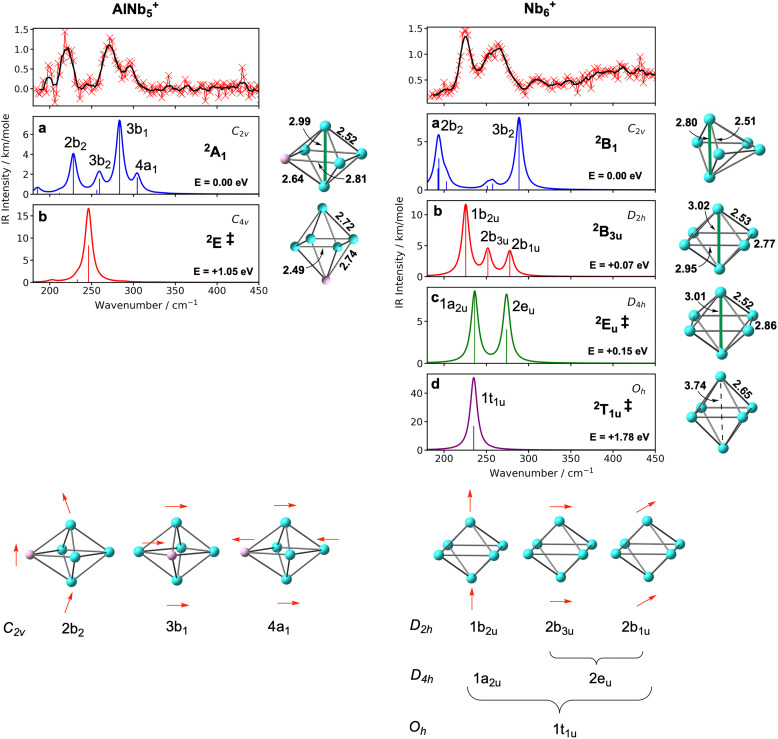
Measured IRMPD spectra of AlNb_5_^+^ ·Ar (left panel) and Nb_6_^+^·Ar (right panel) complexes and DFT computed vibrational spectra. The short *trans*-annular Nb-Nb separation is highlighted by a green line in the structural diagrams, but this does not imply the presence of a direct Nb–Nb bond (see SI for details). The computed spectra are presented for a number of low-energy isomers, and the atomic motions associated with the most intense vibrational modes are visualized below. The descent in symmetry from *O*_h_ to *D*_4h_ to *D*_2h_ for Nb_6_^+^ is shown in the right panel. A full discussion of the electronic structure of this cluster is presented in supporting information. ‡ indicates a first or higher-order saddle point.

The spectra of AlNb_5_^+^ and Nb_6_^+^ are reproduced in the left and right panels of Fig. 2, respectively, along with a series of DFT-computed spectra for various candidate structures. Structures and spectra for higher-energy isomers are collected in the Fig. S5, S6 and S7. The spectra are largely featureless between 450 and 600 cm^−1^, so the *x* axis is truncated at 450 cm^−1^. The all-Nb cluster, Nb_6_^+^, has been studied extensively by Fielicke and co-workers,^19^ and the spectrum reported here is very similar to that shown in Fig. 3 of ref. 19, with an intense band at 226 cm^−1^ and a broader one at 266 cm^−1^. Based on a good agreement between the experimental and computed spectra over the 85–400 cm^−1^ window, the equilibrium structure was assigned to a *D*_2h_-symmetric distorted octahedron, identified as isomer b, with a ^2^B_3u_ ground state, in Fig. 2. This isomer was identified as the global minimum in reference,^19^ lying 0.12 eV below a *C*_2v_-symmetric dimer-capped rhombus, ^2^B_1_ (isomer a in Fig. 2). Our own calculations with the PBE functional reverse the energetic order of the two states, placing isomer a 0.07 eV below isomer b. The energetic difference is, however, marginal, and the much better match to the experimental spectrum afforded by isomer b leads us to adopt Fielicke's assignment of the *D*_2h_-symmetric isomer as the equilibrium structure proposed in ref. 19.

The *D*_2h_-symmetric optimised structure of Nb_6_^+^ shows a very pronounced compression along one axis of the distorted octahedron, leading to a strikingly short *trans*-annular Nb–Nb distance of 3.02 Å, only marginally longer than the bond lengths around the equator of the cluster, and also an asymmetry in the Nb–Nb bonds around the equatorial plane (2.95 Å and 2.77 Å). It is beyond the scope of this paper to give a detailed analysis of the electronic origins of these structural distortions; however they are intimately connected to the near-degeneracy of the orbitals in the approximately half-filled 4d band of Nb (see SI for a more detailed discussion). It is important to emphasise that the short *trans*-annular distance in Nb_6_^+^ does not imply the presence of a direct Nb–Nb bond between these atoms – indeed there is no evidence from a Quantum Theory of Atoms in Molecules (QTAIM) analysis for such a bond (SI). Rather, the bonds between the apical Nb atoms and the four equatorial atoms are stronger than those around the equator (2.53 Å *vs.* 2.77 Å and 2.95 Å), which compresses the *trans*-annular distance indirectly. A discussion of the electronic structure of the wider family of M_*n*_ will be presented in a subsequent paper; in this paper we focus on the implications of the distortions for the vibrational spectra.

There are three vibrational bands of significant intensity for isomer **b** (^2^B_3u_ ground state), labelled 1b_2u_, 2b_3u_ and 2b_1u_ in Fig. 2, which correspond to rigid translations of the *trans*-annular Nb_2_ unit in three orthogonal directions (see depictions of the modes in Fig. 2). The 1b_2u_ mode involves motion of the Nb_2_ unit perpendicular to the equatorial plane, while the less intense 2b_3u_ and 2b_1u_ bands at 252 cm^−1^ and 278 cm^−1^, respectively, correspond to oscillations in the plane: the 2b_3u_ and 2b_1u_ modes are collectively assigned to the broad experimental band around 266 cm^−1^. If perfect *D*_4h_ symmetry is imposed (isomer c, a 1st-order saddle point), the 2b_3u_ and 2b_1u_ modes merge into a degenerate 2e_u_ mode, while in a perfect octahedron (isomer d, a 3rd-order saddle point), the three bands 1b_2u_, 2b_3u_ and 2b_1u_ merge into a single intense t_1u_-symmetric mode at 235 cm^−1^. The splitting of the intense bands in Nb_6_^+^ is therefore a sensitive probe of the extent to which the geometry deviates from a perfect octahedron. The relative energies of the *O*_h_, *D*_4h_ and *D*_2h_ isomers (+1.78 eV, +0.15 eV, +0.07 eV, respectively) confirm that in energetic terms, the axial compression is by far the most significant, and indeed we show in the following sections that it is a persistent structural motif through all of the 6-, 7- and 8-vertex clusters.

In addition to the three sharp bands below 300 cm^−1^ that were discussed in the previous paragraph, the spectrum of Nb_6_^+^ also has a broad feature between 350 and 500 cm^−1^ that is not present in any of the other clusters. The corresponding spectrum reported by Fielicke and co-workers was truncated at 400 cm^−1^,^19^ but there also, the low-frequency tail of the broad band can be identified just below 400 cm^−1^. The highest of the fundamental vibrations is a dipole-forbidden (a_g_) breathing mode at 317 cm^−1^, well below the onset of the broad absorption feature. It is possible that this broad band could be assigned to an overtone or combination band or, alternatively, to a low-lying electronic transition such as the one identified by Bakker and co-workers at 476 cm^−1^ in the spectrum of the tantalum carbide cluster Ta_5_C_3_.^42^ A number of recent studies have also emphasised the importance of low-lying electronic states in open-shell cobalt clusters, Co_*n*_^+^.^43,44^ In the context of Nb_6_^+^, we note that the *D*_2h_-symmetric minimum is only 0.08 eV below the *D*_4h_-symmetry saddle point, so at finite temperature, the cluster is likely to be highly dynamic, sampling a range of geometries around the high-symmetry structure, where the HOMO–LUMO gap vanishes. The near degeneracy of the orbital manifold is therefore fully consistent with the possible presence of a low-lying electronically excited state.

The IR-MPD spectrum of AlNb_5_^+^ is strikingly similar to that of Nb_6_^+^, with two bands of significant intensity at 222 cm^−1^ and 272 cm^−1^, the latter with a distinct shoulder at 296 cm^−1^. Less intense features also appear at 200 cm^−1^ and 244 cm^−1^. Our survey of the potential energy surface reveals that the lowest-energy isomer, isomer a (^2^A_1_ ground state), has *C*_2v_ point symmetry, with the Al atom occupying one equatorial vertex of a distorted octahedron, where it is bound much more strongly to the axial Nb atoms (2.64 Å) than it is to the equatorial ones (2.81 Å). Isomer a inherits the axial compression in Nb_6_^+^, with a *trans*-annular Nb–Nb separation of 2.99 Å, compared to 3.02 Å in the all-Nb analogue. The alternative arrangement with *C*_4v_ symmetry, isomer b, where the Al occupies an axial site in the compressed octahedron, is a saddle point and is over 1 eV less stable. The computed spectrum of isomer a offers a compelling match to the experiment, with two intense bands, 2b_2_ and 3b_1_, at 228 cm^−1^ and 283 cm^−1^, respectively and a less intense transition, 4a_1_, at 305 cm^−1^ that we assign to the shoulder at 296 cm^−1^ in the experimental spectrum. A second relatively weak transition at 259 cm^−1^, 3b_2_, may correspond to the small peak at 244 cm^−1^. We can again associate the intense features with the motion of the Nb_2_ unit: the 2b_2_, 3b_1_ modes are the direct analogues of 1b_2u_ and 2b_3u_, respectively in Nb_6_^+^.

### AlNb_6_^+^ and comparison with Nb_7_^+^

**Fig. 3:**
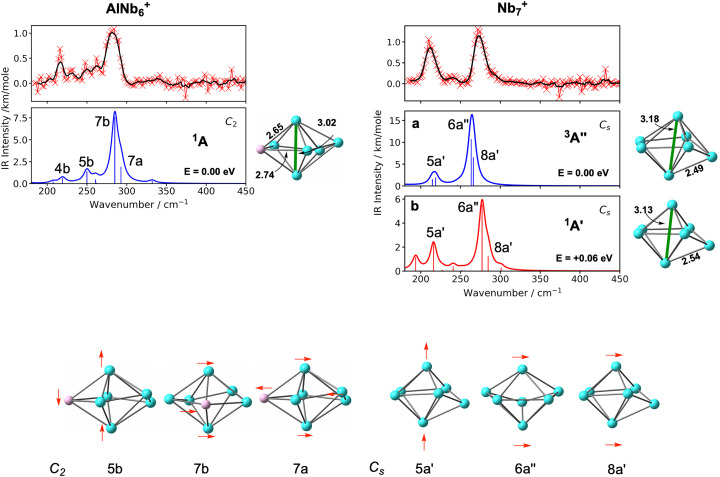
Measured IRMPD spectra of AlNb_6_^+^·Ar (left panel) and Nb_7_^+^·Ar (right panel) complexes and DFT computed vibrational spectra. The computed spectra are presented for a number of low-energy isomers, and the atomic motions associated with the most intense vibrational modes are visualized below. The similarity between the intense modes and those in Fig. 2 should be noted.

The spectra of AlNb_6_^+^ (Fig. 3, left panel) and Nb_7_^+^ (right panel) again show a striking resemblance, particularly in the 250–300 cm^−1^ window. The major features of Nb_7_^+^ are almost identical to those reported in ref. 19, with two bands with significant intensity at 212 cm^−1^ and 272 cm^−1^, along with a smaller feature around 240 cm^−1^. Fielicke and co-workers have assigned the equilibrium structure as a singlet state with a distorted pentagonal bipyramidal geometry.^19^ A triplet with similar structure was reported to be 0.47 eV higher in energy, and was rejected on that basis and also because it offered a poorer match to experiment across the entire 85–400 cm^−1^ range available in their experiments. Like Nhat and co-workers,^21^ we find the triplet (isomer a) to be marginally more stable than the singlet (isomer b) with the PBE functional, although Nhat also noted that the singlet is relatively stabilized at the CCSD(T) level, such that it becomes the ground state. For these reasons, we focus our normal mode analysis on isomer b in Fig. 3, despite the fact that we compute it to be marginally less stable than the triplet; the spectra of singlet and triplet are in any case very similar in the 200–450 cm^−1^ range. The distorted pentagonal bipyramidal structure of isomer b again shows evidence for an axial compression, with a short *trans*-annular Nb–Nb distance of 3.13 Å. The computed spectrum features an intense transition, 6a″, at 277 cm^−1^ with a smaller feature, 8a′, forming a shoulder to the high-frequency side of the main band. Collectively, these two transitions are assigned to the broad peak in the experimental spectrum at 272 cm^−1^, and they are closely related to the 2b_3u_ and 2b_1u_ modes of Nb_6_^+^, in so much as the *trans*-annular Nb_2_ unit is moving perpendicular to the axis connecting the two atoms. The 5a′ mode at 216 cm^−1^ is assigned to the intense peak at 212 cm^−1^, and is the direct analogue of the 1b_2u_ mode in Nb_6_^+^ and the 2b_2_ mode in AlNb_5_^+^.

The most stable isomer of AlNb_6_^+^, isomer a, proves to be a substitutional isomer of Nb_7_^+^, a distorted pentagonal bipyramid with *C*_2_ symmetry, with the Al atom occupying one of the positions in the pentagonal plane. The axial compression that was present in Nb_6_^+^ and AlNb_5_^+^ is again a feature, and the two apical Nb atoms of the pentagonal prism are separated by only 3.02 Å (*vs.* 2.99 Å in AlNb_5_^+^). The Al atom is bonded more strongly to the axial Nb atoms than it is to their equatorial counterparts (Al–Nb = 2.65 Å and 2.74 Å, respectively), although the distinction is not as great as it is in AlNb_5_^+^. The computed vibrational spectrum is dominated by one intense peak, 7b which matches the feature in the experimental spectrum at 282 cm^−1^. The 7b mode corresponds to motion of the Nb_2_ unit perpendicular to its own axis, and is similar, in terms of frequency, intensity and amplitude, to the 6a″ mode in Nb_7_^+^, the 2b_3u_ and 2b_1u_ modes of Nb_6_^+^ and the 3b_1_ mode of AlNb_5_^+^, all of which are the most intense feature in their respective spectra. The 4b mode, possibly responsible for the small absorption feature at 216 cm^−1^, corresponds to the motion of the Nb_2_ unit perpendicular to the pentagonal plane, and is the direct analogue of 2b_2_ in AlNb_5_^+^ which appears at 228 cm^−1^, albeit with much greater intensity. We also note here that the Nb_6_^+^ fragment in the *C*_2_-symmetric ground state bears a striking resemblance to the *C*_2v_-symmetric isomer of Nb_6_^+^, the dimer-capped rhombus discussed in the previous section. This structural resemblance extends to the spectrum of this isomer, which is also dominated by a single mode, 3b_2_ in Fig. 2.

### AlNb_7_^+^ and comparison with Nb_8_^+^

**Fig. 4:**
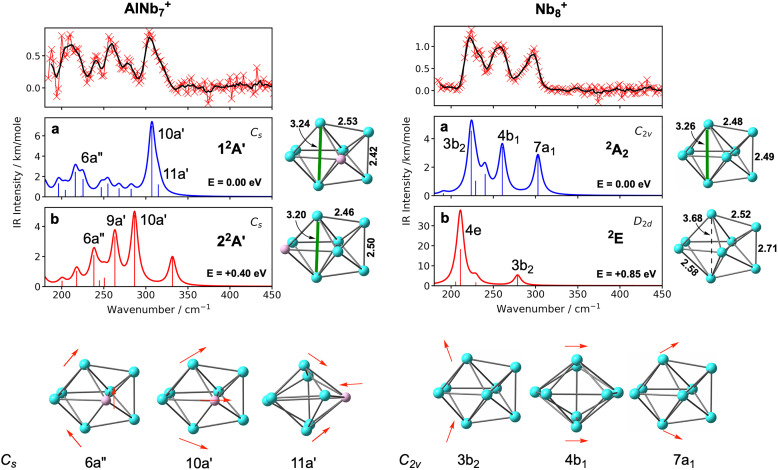
Measured IRMPD spectra of AlNb_7_^+^·Ar (left panel) and Nb_8_^+^·Ar (right panel) complexes and DFT computed vibrational spectra. The computed spectra are presented for a number of low-energy isomers, and the atomic motions associated with the most intense vibrational modes are visualized below.

Unlike the 6- and 7-vertex clusters discussed in the previous sections, the correspondence between the spectra of AlNb_7_^+^ and Nb_8_^+^ is less obvious. The spectrum of Nb_8_^+^ shown in Fig. 4 is again very similar to that reported by Fielicke and co-workers,^19^ with three peaks of similar intensity at 222, 258 and 298 cm^−1^. The 258 cm^−1^ band is broad, but there is no evidence for the distinct splitting of this band reported in the previous work (see Fig. S2).^19^ This subtle difference is most likely a consequence of a narrow emission gap at 39 µm in the source used in the earlier experiments. Our calculations also concur with the previous work in identifying the most stable isomer of Nb_8_^+^ as a *C*_2v_-symmetric structure (isomer a, ^2^A_2_ ground state), a bi-capped octahedron. An ideal bi-capped octahedron (also known as a snub-disphenoid) would have *D*_2d_ symmetry, but in the optimised structure we again see a compression along one of the *trans*-annular Nb–Nb axes (Nb–Nb = 3.26 Å), which lowers the symmetry to *C*_2v_. The energetic significance of the distortion is again made clear by imposing *D*_2d_ point symmetry (isomer b), which forces the *trans*-annular Nb–Nb distances to be equal: this structure (^2^E in Fig. 4) is 0.85 eV higher in energy. The computed spectrum of the ^2^A_2_ isomer offers a strong match to experiment in the 200–310 cm^−1^ window: the peaks at 222, 258 and 298 cm^−1^ can be assigned to 3b_2_, 4b_1_ and 7a_1_, respectively, again corresponding to motion of the *trans*-annular Nb_2_ unit in three orthogonal directions. The 261 cm^−1^ and 303 cm^−1^ bands (4b_1_ and 7a_1_, respectively) are the analogues of the near-degenerate 2b_3u_, 2b_1u_ pair in Nb_6_^+^, now split by 42 cm^−1^ due to the more pronounced prolate geometry of the Nb_8_^+^ cluster compared to Nb_6_^+^. The lowest frequency mode, 3b_2_, is the direct analogue of 1b_2u_ in Nb_6_^+^ and 5a′ in Nb_7_^+^.

The most intense peak in the measured spectrum of AlNb_7_^+^ occurs at 304 cm^−1^, with a broad shoulder to higher frequency: this marks a distinct change compared to AlNb_5_^+^ and AlNb_6_^+^, where the highest-frequency intense peak was found below 300 cm^−1^. Peaks with somewhat lower intensity are also found at 212 cm^−1^ and 260 cm^−1^. The lowest energy isomer shown in Fig. 4 proves to be a doublet with *C*_s_ point symmetry, isomer a (^2^A′ ground state), where the Al atom replaces a Nb at one of the equatorial sites in the bi-capped octahedron of Nb_8_^+^, and the characteristic axial compression across one Nb_2_ pair is retained, albeit rather less pronounced than in the smaller clusters (Nb–Nb = 3.24 Å). Moreover, the Al atom now caps a pentagonal face, rather than a square face as it did in AlNb_5_^+^ and AlNb_6_^+^, and all five Al–Nb bonds are short (2.72–2.75 Å). The next most stable isomer, isomer b (also ^2^A′), where the Al occupies an equatorial site at the pole of the cluster, is 0.4 eV less stable. The computed spectrum of the lowest energy isomer a, shows a single band of high intensity, 10a′ at 307 cm^−1^, coincident with the intense peak in the experimental spectrum. There is also a lower intensity peak at 315 cm^−1^ (11a′) that is consistent with the shoulder on the high-frequency side of the intense band. The region between 220 and 250 cm^−1^ contains a number of transitions of relatively low intensity. The features in the high-frequency region (10a′ and 11a′) can be traced to corresponding bands in Nb_8_^+^: 10a′ is the counterpart of 7a_1_, while 11a′ is the counterpart of 4b_1_, but both are significantly blue shifted in AlNb_7_^+^ compared to AlNb_5_^+^ and AlNb_6_^+^ because the internal modes of the Nb_7_ unit are increasingly strongly coupled to the motion of the lighter Al atom. The fact that modes above 300 cm^−1^ are not as prominent in the smaller clusters appears to reflect a trend towards ‘surface absorption’ of the Al atom *via* many Al–Nb bonds of approximately equal strength (five in this case) as opposed to integration of Al into the core of the cluster *via* two stronger bonds (as in AlNb_5_^+^ and AlNb_6_^+^).

### AlNb_8_^+^ and comparison to Nb_9_^+^

The spectrum of the Nb_9_^+^ cluster has two intense features above 270 cm^−1^, with a number of smaller peaks in the 200–250 cm^−1^, window, and Fielicke's previous work revealed no additional transitions of significant intensity below 200 cm^−1^.^19^ The most stable isomer is a *C*_2_-symmetric singlet where a single Nb atom caps the hexagonal face of a Nb_8_ ‘boat’. The point symmetry is, in fact, very close to *C*_2v_, but a small imaginary frequency in this higher symmetry leads to a rotation of the Nb_2_ unit at the base of the boat. The short *trans*-annular Nb–Nb separation that featured in all of the smaller clusters is absent in Nb_9_^+^, the shortest such distance being 3.40 Å, signaling a discontinuity in the growth pattern. The computed vibrational spectrum shows intense features at 269 cm^−1^ (9b) and 291 cm^−1^ (10b) which correspond closely to the two peaks in the experimental spectrum.

**Fig. 5:**
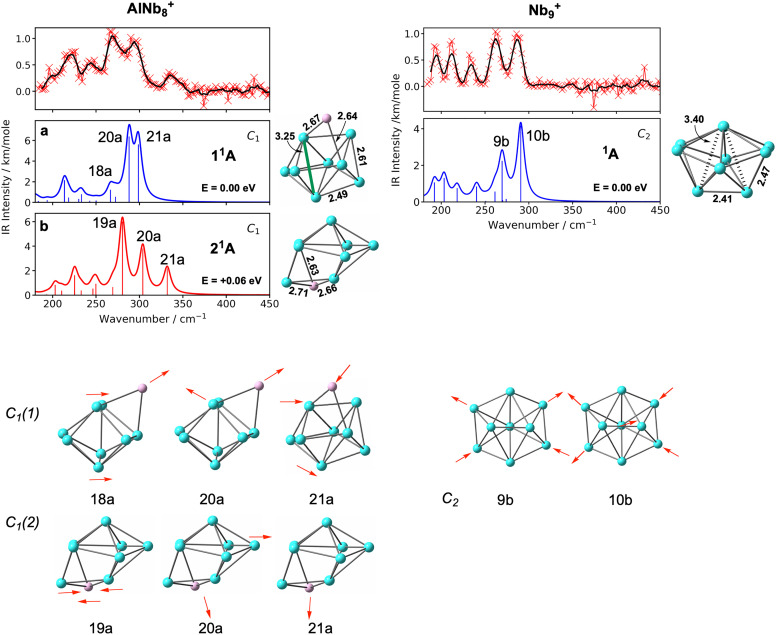
Measured IRMPD spectra of AlNb_8_^+^·Ar (left panel) and Nb_9_^+^·Ar (right panel) complexes and DFT computed vibrational spectra. The computed spectra are presented for a number of low-energy isomers, and the atomic motions associated with the most intense vibrational modes are visualized below. For AlNb_8_^+^, the less stable isomer b offers a better match to experiment than isomer b, particularly in the high-frequency region around 340 cm^−1^.

The spectrum of AlNb_8_^+^ has two intense bands between 268 and 294 cm^−1^, a smaller but distinct feature at 336 cm^−1^ and a broad absorption feature in the 200–250 cm^−1^ window. We have identified two almost degenerate local minima on the potential energy surface, isomer a and isomer b in Fig. 5. The more stable of these, isomer a, has a Nb_8_^+^ unit rather similar to the structure of Nb_8_^+^ itself, with an Al atom capping a triangular Nb_3_ face. The Nb_8_ core is perturbed little by the presence of the Al cap – in particular, the *trans*-annular Nb–Nb distance of 3.25 Å is identical to that in Nb_8_^+^. Isomer b, in contrast, has a different Nb_8_ core, based on a capped pentagonal bipyramid. The Al atom caps a rhombic Nb_4_ face with four Al–Nb bond lengths between 2.62 and 2.94 Å. The two isomers are separated by only 0.06 eV – too close to warrant a definitive judgement on the identity of the dominant species present in the gas phase – so we rely heavily on a comparison of the experimental and computed spectra. Both isomers generate intense absorptions in the 270–300 cm^−1^ window, where there are two prominent bands in the experimental spectrum. However, only isomer b has a band of significant intensity above 320 cm^−1^ (21a, 332 cm^−1^), which corresponds to the motion of Al perpendicular to the rhombic face. On that basis, we argue that isomer b is the most probable candidate for the structure present in the gas phase, despite the fact that it is computed to be marginally higher in energy.

### AlNb_9_^+^ and comparison Nb_10_^+^

**Fig. 6:**
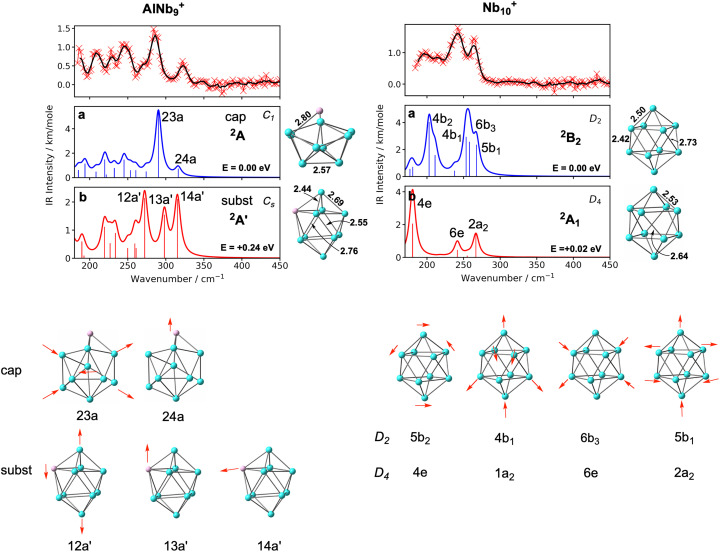
Measured IRMPD spectra of AlNb_9_^+^·Ar (left panel) and Nb_10_^+^·Ar (right panel) complexes and DFT computed vibrational spectra. The computed spectra are presented for a number of low-energy isomers, and the atomic motions associated with the most intense vibrational modes are visualized below. Note the close resemblance, both structural and spectroscopic, between the ‘cap’ isomer of AlNb_9_^+^ and Nb_9_^+^ shown in Fig. 5, particularly in the amplitudes of the 23a and 10b modes, respectively.

In the spectrum of Nb_10_^+^, shown in Fig. 6, an intense feature appears at 242 cm^−1^, with a second band of lower intensity at 264 cm^−1^ and a broad absorption between 190 and 220 cm^−1^, very similar to the spectrum reported by Fielicke and Meijer in 2011.^25^ At that time, the authors proposed a *D*_4_-symmetric structure, a distorted bicapped square antiprism, for this cluster, although they noted that a high-intensity band predicted at 172 cm^−1^ for this isomer was conspicuously absent from the measured spectrum.^25^ We have re-explored the potential energy surface using the PBE functional, and identified this *D*_4_-symmetric structure (isomer b): its ground state has ^2^A_1_ symmetry, and we calculate the same intense *e*-symmetric vibrational mode at 180 cm^−1^ (4e in Fig. 6), corresponding to a wagging motion of the apical atoms. We have, however, also identified a *D*_2_-symmetric structure, isomer a, that is 0.02 eV lower in energy, where the apical Nb atoms are bonded more strongly to two members of the square face than the others (Nb–Nb = 2.49 Å and 2.61 Å *vs.* 2.53 Å in the *D*_4_-symmetric structure). The *D*_2_ symmetry distortion splits the problematic *e*-symmetry mode at 180 cm^−1^ into two components, one at 203 cm^−1^ (4b_2_ in Fig. 6), which coincides with a broad absorption feature in the experimental region, and another, 1b_3_ at 116 cm^−1^, leaving the window around 180 cm^−1^ free of intense absorptions. The better correspondence to experiment, and the absence of any other low-energy isomers, suggests that the distorted bi-capped square antiprism is indeed the dominant isomer in the gas phase.

The spectrum of the AlNb_9_^+^ cluster immediately appears rather different to Nb_10_^+^: the most intense band appears at 286 cm^−1^, and there is a small but very distinct feature at 322 cm^−1^, similar to the spectrum of AlNb_8_^+^. In our search for low-energy isomers, we have again followed the dual strategy of substituting vertices of Nb_10_^+^ and capping faces and edges of Nb_9_^+^, and we have located relatively stable local minima that fall into both categories. In this case, the more stable isomer (isomer a in Fig. 6) also offers the best match to the measured spectrum. The Al atom caps a rhombic Nb_4_ face of the Nb_9_ unit, the geometry of which is almost identical to the Nb_9_^+^ cluster shown in Fig. 5. The single intense peak in the calculated spectrum, 23a, is also remarkably similar in frequency, intensity and amplitude to the 10b mode of Nb_9_^+^, with minimal amplitude on the Al atom. The high-frequency mode, 24a, is reminiscent of the 21a mode of the second isomer of AlNb_8_^+^, where the Al atom also caps a rhombic Nb_4_ face. Both modes correspond to motion of the Al atom perpendicular to this Nb_4_ face, with very little amplitude on the Nb_8_ or Nb_9_ units. The appearance of a band in the 320–330 cm^−1^ window in the Al/Nb clusters appears, therefore, to be diagnostic of an Al atom capping a rhombic Nb_4_ face. The second isomer, isomer b in Fig. 6, is obtained by Al/Nb substitution of the Nb_10_^+^ structure, and has the Al atom in one of the equatorial positions, rather than along the principal axis of the bicapped square antiprism. This isomer is 0.24 eV higher in energy than the capping isomer, and offers a rather poorer match to the experiment.

### Cluster stability and Al binding energies

Our analysis of the vibrational spectra of the family of AlNb_*n*_^+^ clusters suggests a distinct transition from Al/Nb substitution of Nb_*n*+1_^+^ in the smaller members to surface binding of Al to a rhombic face of Nb_*n*_^+^ in the larger members. We can define the binding energies of Nb and Al to the Nb_*n*_^+^ clusters as follows:*E*(Nb) + *E*(Nb_*n*_^+^) → *E*(Nb_*n*+1_^+^)*E*(Al) + *E*(Nb_*n*_^+^) → *E*(AlNb_*n*_^+^)

**Fig. 7:**
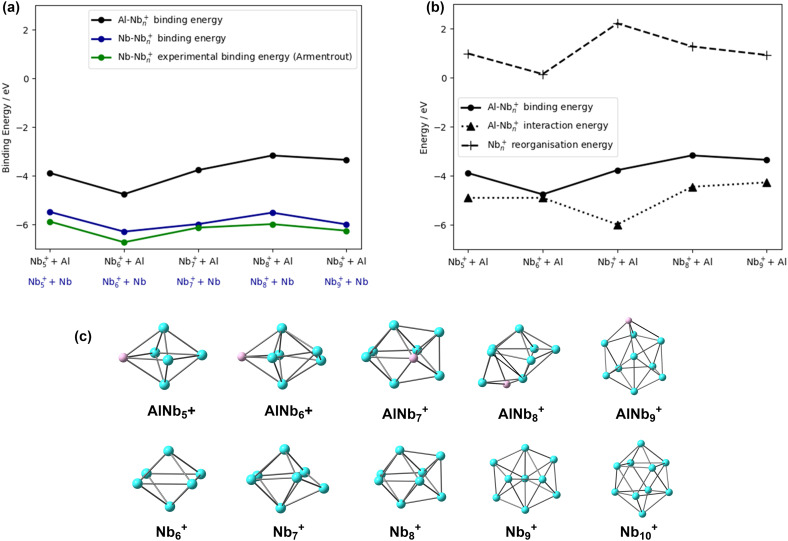
(a) Al and Nb binding energies in AlNb_*n*_^+^ and Nb_*n*+1_^+^; the computed total binding energies are shown in black and blue circles, respectively, while the corresponding experimental data for Nb_*n*_^+^^45^ is shown in green. (b) Decomposition of the Al-binding energy (black circles) into the re-organisation energy of the Nb_*n*_^+^ fragment (black crosses) and the interaction energy for the Al atom with the deformed fragment (black triangles). (c) Structures of the AlNb_*n*_^+^ and Nb_*n*+1_^+^ clusters.

The computed binding energies for Al (black) and Nb (blue) as a function of cluster size are shown in Fig. 7(a), where the experimental data for the all-Nb series measured by Armentrout is shown for comparison (green).^45,46^ For Nb_*n*_^+^, the DFT calculations (blue) reproduce the trends in the experimental data (green), with average values around 6 eV (compared to a cohesive energy of 7.07 eV per atom in bulk Nb). The calculations also reproduce the strong binding in Nb_7_^+^ compared to both Nb_6_^+^ and Nb_8_^+^. The Al binding energies (black) track the same overall trend, with Al binding also strongest in the 7-vertex cluster AlNb_6_^+^, −4.76 eV. The binding of Al is, however, on average more than 2 eV less favourable than the binding of an additional Nb atom, and this naturally leads to an increasing tendency for the Nb atoms to bond to each other as the cluster grows. There is therefore a trend towards surface binding of Al, where the strong Nb–Nb bonding is disrupted least, in the larger clusters. The strong Nb–Nb bonding will also disfavour endohedral encapsulation of the Al atom, and indeed Pansini *et al.* have reported no evidence for such encapsulation even with 10 Nb atoms.^17^

In Fig. 7(b), the Al binding energy is broken down into the sum of two terms, the reorganisation energy required to deform the Nb_*n*_^+^ cluster from its equilibrium geometry to the geometry it adopts in AlNb_*n*_^+^ (crosses in Fig. 7(b)), and the Al interaction energy, the binding of Al to this deformed fragment (triangles). In all cases, the energy of the Nb_*n*_^+^ cluster corresponds to the spin state found in its equilibrium geometry, and the energy of the Al atom corresponds to its doublet ground state (3s^2^3p^1^). The energy decomposition shows that the very strong binding of Al in AlNb_6_^+^ (and, by extension, of Nb in Nb_7_^+^) is a direct consequence of a very small re-organisation energy (+0.15 eV) which is, in turn, closely linked to the presence of the two near-degenerate isomers with *D*_2h_ (isomer b) and *C*_2v_ (isomer a) symmetry in Fig. 2. The malleability of the Nb_6_^+^ core then allows an additional atom to be incorporated with relatively little cost in terms of Nb–Nb bonding. At the opposite extreme, the binding energy in AlNb_7_^+^ is relatively low because the re-organisation energy is high (+2.22 eV): the Nb_7_^+^ fragment must undergo a significant rearrangement to create a vacancy at the equatorial site of the compressed octahedron. The reorganisation energy is then compensated to some extent by strong Al–Nb bonding to a pentagonal face, but the net result is still weaker Al binding than in AlNb_6_^+^. For the AlNb_8_^+^ and AlNb_9_^+^ clusters, the relatively weak binding of Al arises from a combination of low re-organisation energies but also low interaction energies, consistent with a transition from substitution to surface binding to a rhombic face. The lower Al–Nb_*n*_^+^ interaction energies for the larger clusters correlate with the emergence of features above 300 cm^−1^ in the vibrational spectra.

## Summary and conclusions

In this paper, we have reported the IR-MPD spectra of a family of Al/Nb clusters, AlNb_*n*_^+^, and drawn comparison to their all-Nb analogues, Nb_*n*+1_^+^. Complementary DFT calculations are used to identify the most stable isomers in the two families, using both the calculated energies and the match between experiment and theory as criteria. The results reveal very different growth patterns in the small and large clusters, with a discontinuity occurring between AlNb_7_^+^ and AlNb_8_^+^. In the small clusters (AlNb_5_^+^, AlNb_6_^+^ and AlNb_7_^+^), the most stable isomer is obtained by replacing one vertex of the corresponding all-Nb cluster with the same number of vertices (*i.e.* AlNb_5_^+^ is obtained by Al/Nb substitution at one vertex of the Nb_6_^+^ cluster). The Nb_6_^+^ cluster has a distorted octahedral core with a very short *trans*-annular separation, which proves to be a common feature of the clusters with 6, 7 or 8 atoms, and all of their spectra can then be assigned based on the rigid translations of a Nb_2_ dumbbell in three orthogonal directions. The structures of the larger clusters (AlNb_8_^+^, AlNb_9_^+^), in contrast, are derived by capping the equilibrium structures of Nb_8_^+^ and Nb_9_^+^ with an Al atom on a rhombic face, and the spectra therefore bear closer resemblance to the all-Nb analogues with the same number of Nb atoms, with additional low-intensity features in the 320–330 cm^−1^ window reflecting the presence of the surface-bound Al atom. Our findings are therefore consistent with Nonose's studies of the reactivity of neutral AlNb_*n*_ with H_2_,^6^ where they concluded that the Al atoms blocked highly active sites in the smaller clusters (sites in the compressed octahedron), but were absorbed on the surface in the larger clusters.

## Author contributions

Conceptualisation: PF, EJ, JEM and PL. Investigation: PF, PL, RS, DP, JMB. Writing – original draft: RS. Formal analysis: PF, RS. Supervision: EJ, JEM, PL. Writing – review and editing: RS, AF, EJ, JEM, PL, PF. Supervision: funding acquisition: EJ, JEM and PL.

## Conflicts of interest

There are no conflicts of interest to declare.

## Supplementary Material

CP-028-D6CP01125J-s001

## Data Availability

The data supporting this article have been included as part of the Supplementary Information (SI). These include a list of total energies and cartesian coordinates of all reported minima and a discussion of the electronic structure of Nb_6_^+^. See DOI: https://doi.org/10.1039/d6cp01125j.
