# Supplementary material for: Vibrational spectroscopy, structure and bonding in the family of aluminium-doped niobium clusters, AlNbn+, n = 5–9
Source: Phys Chem Chem Phys. 2026 Jul 3;28(30):18541–52. doi: 10.1039/d6cp01125j (PMC13367229; doi:10.1039/d6cp01125j)
Supplement: CP-028-D6CP01125J-s001 [file CP-028-D6CP01125J-s001.pdf]

Supplementary Information (SI) for Physical Chemistry Chemical Physics.
This journal is © the Owner Societies 2026

Vibrational spectroscopy, structure and bonding in the family of aluminium-doped niobium clusters, $AlNb_n^+$, $n = 5 - 9$.

Supporting Information

Roshan Singh,[a] Piero Ferrari,[b,d‡] Deepak Pradeep,[b‡] Joost M. Bakker, [b‡] André Fielicke,[c‡] Ewald Janssens,[d‡] Peter Lievens,[d‡] John E. McGrady.[a]

[a] *Department of Chemistry, University of Oxford, South Parks Road, Oxford OX1 3QR, U.K.*

[b] *HFML-FELIX, Institute for Molecules and Materials, Radboud University, Toernooiveld 7 6525 ED, Nijmegen, The Netherlands; piero.ferrariramirez@ru.nl.*

[c] *Fritz-Haber-Institut der Max-Planck-Gesellschaft, Faradayweg 4-6, 14195 Berlin, Germany.*

[d] *Quantum Solid-State Physics, Department of Physics and Astronomy, KU Leuven, Celestijnenlaan 200 D, B-3001 Leuven, Belgium.* E-mail: piero.ferrariramirez@ru.nl, ewald.janssens@kuleuven.be, john.mcgrady@chem.ox.ac.uk

Contents

1 Mass spectrum of Nb_n^+ (n=5-10) and $AlNb_m^+$, (m=5-9) clusters 3

2 Comparison of Nb_n^+ (n=6-10) IRMPD spectra measured *via* FELIX vs FELICE 4

3 Influence of the Ar tag on computed spectra. 7

4 Detailed Survey of the Potential Energy Landscape, including higher energy structures than those shown in the main text. 8

5 Electronic Structure of the Nb_6^+ Cluster. 11

6 Total energies and XYZ coordinates 15

7 Sample ADF input file 26

List of Figures

S1 Mass spectrum of Al doped Nb clusters produced at 200 K with 2% Ar in the He carrier gas. 3

S2 Comparison of the IR-MPD spectra for Nb_n^+ (n=6-10) clusters measured using FELIX (left) and FELICE (right). Note the broader bands in the FELICE measurements. This is a result of higher IR fluence over a larger interaction volume, which produces more efficient multiphoton absorption and leading to power broadening of the spectral features compared to FELIX. 5

S3 Computed spectra in the 85-400 cm^{-1} range for the proposed ground state structures of Nb_n^+ (n=6-10) clusters. 6

S4 Comparison of spectra of $AlNb_n^+$ with (red) and without (blue) explicit inclusion of Ar tags in the computational model 7
S5 Measured IRMPD spectra of $AlNb_n^+{\cdot}Ar$ and $Nb_n^+{\cdot}Ar$ complexes and DFT computed vibrational spectra. The relative energies, spin state and structures are also presented. 9
S6 Measured IRMPD spectra of $AlNb_n^+{\cdot}Ar$ and $Nb_n^+{\cdot}Ar$ complexes and DFT computed vibrational spectra. The relative energies, spin state and structures are also presented. 10
S7 Measured IRMPD spectra of $AlNb_n^+{\cdot}Ar$ and $Nb_n^+{\cdot}Ar$ complexes and DFT computed vibrational spectra. The relative energies, spin state and structures are also presented. 11
S8 (a) Orbital symmetries (b) and molecular orbital array for octahedral Nb_6^+, showing evolution in orbital energies as the symmetry is reduced from $O_h \rightarrow D_{4h}$. 14

1 Mass spectrum of Nb_n^+ (n=5-10) and $AlNb_m^+$, (m=5-9) clusters

The mass spectrum of Nb_n^+ (n=5-10) and $AlNb_m^+$ (m=5-9) and their Ar-tagged analogues is shown in Figure S1. The Nb_n^+ and $AlNb_m^+$ clusters are labelled explicitly, while the Ar complexes are marked with an asterisk. The vertical lines represent clusters with 1, 2 and 3 Al atoms

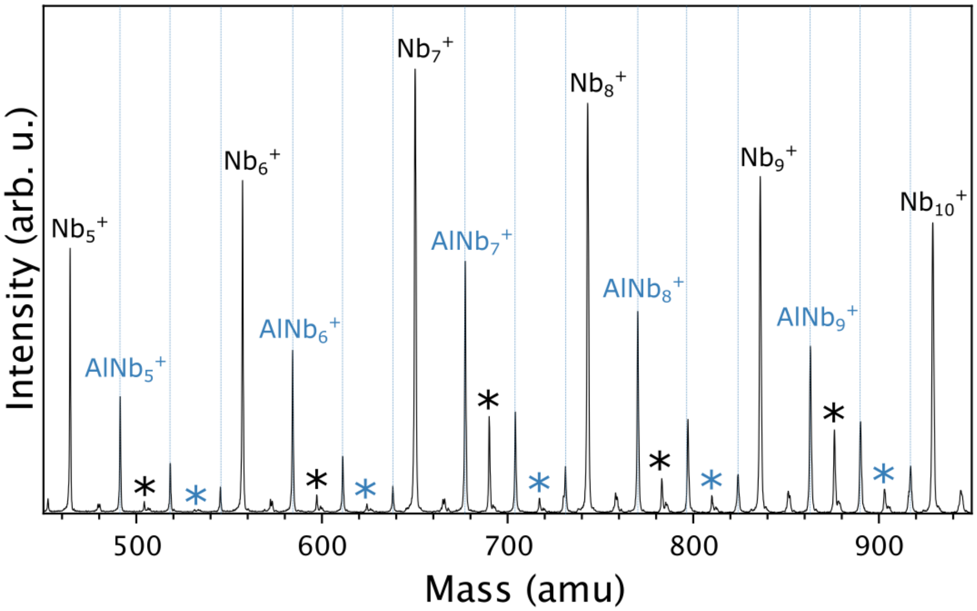

Figure S1 Mass spectrum of Al doped Nb clusters produced at 200 K with 2% Ar in the He carrier gas.

2 Comparison of Nb_n^+ (n=6-10) IRMPD spectra measured *via* FELIX vs FELICE

The measured spectra for the Nb_n^+, $n = 6-10$ clusters in this study are very similar to those reported by Fielicke *et al* in 2007.[1]. The original spectra from Fielicke's work and the new data reported here are compared side-by-side in Figure S2. Note that the low-frequency range of the spectrum (between 85 and 190 cm^{-1}) was not probed in the current work. The signal to noise ratio is generally better in the newer FELICE experiments, due to a hard-focused IR beam (FELIX) but the bands sometimes appear slightly broader. The main difference between the old FELIX spectra compared to the new ones for the bare Nb clusters is a dip in the spectrum of Nb_8^+ at about 39 μm (256 cm^{-1}) that leads to a splitting into 2 peaks, while in the FELICE spectrum only a single peak is seen. This can be understood by an insufficient correction for laser fluence changes in the old FELIX spectra. Specifically the 39 μm gap, where IR power is vanishing in a very narrow range due to a mode-hop in the FEL-1 of FELIX that contained a partial wave-guide, was only understood and accounted for in later experiments.

The spectra reported in the main text are truncated at 190 cm^{-1} to match the new FELICE experimental data, but to afford a comparison over the lower frequency range probed by the older FELIX data, we reproduce the computed spectra down to 85 cm^{-1} in Figure S3. The spectra are largely devoid of intense features below 200 cm^{-1}. This is a significant observation for the lowest energy isomer of Nb_{10}^+, where the previously reported D_4-symmetric isomer had an intense peak at 180 cm^{-1} that was inconsistent with experiment. This issue has been resolved with the identification of the D_2-symmetric structure in Figure S3.

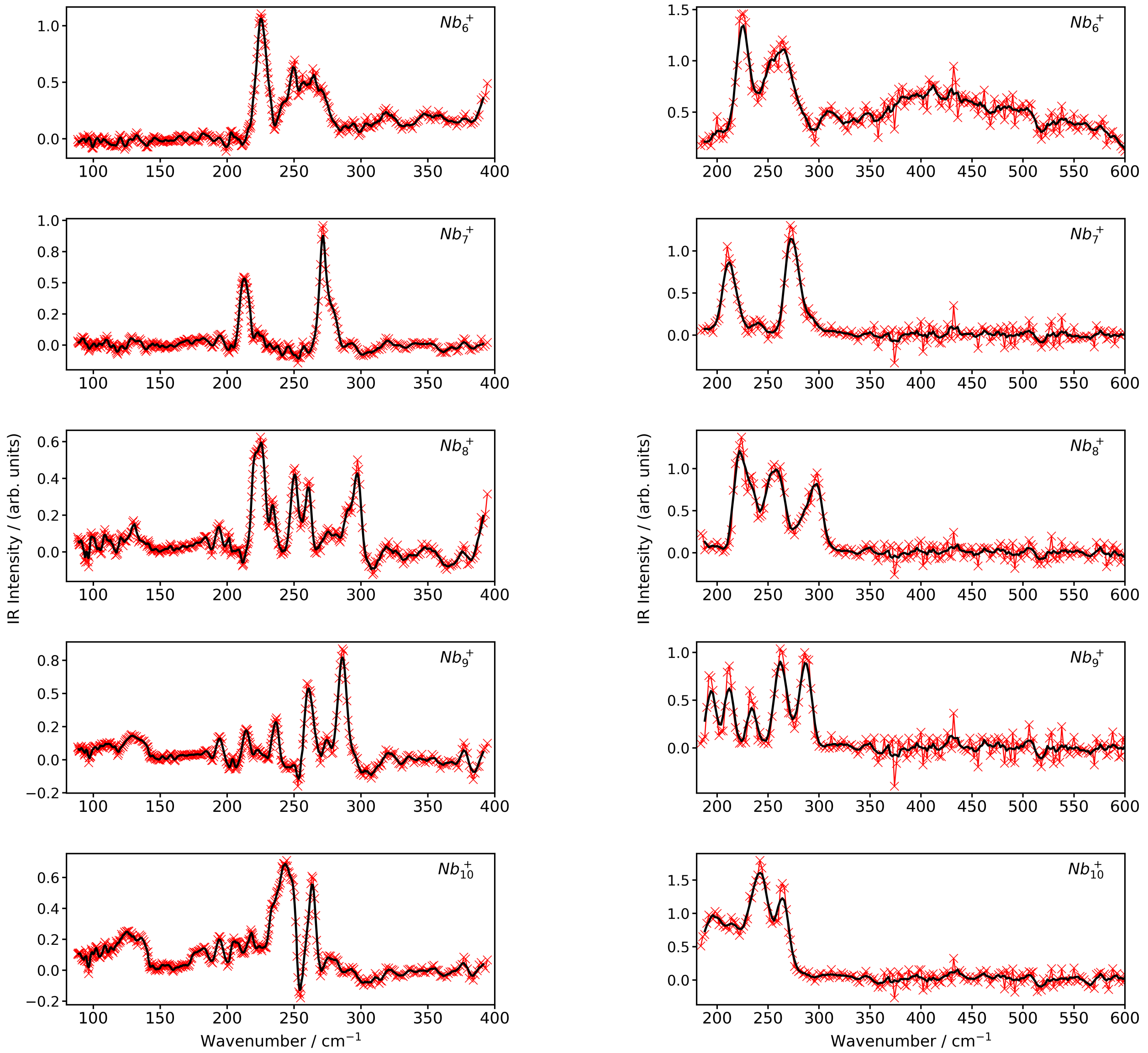

Figure S2 Comparison of the IR-MPD spectra for Nb_n^+ (n=6-10) clusters measured using FELIX (left) and FELICE (right). Note the broader bands in the FELICE measurements. This is a result of higher IR fluence over a larger interaction volume, which produces more efficient multiphoton absorption and leading to power broadening of the spectral features compared to FELIX.

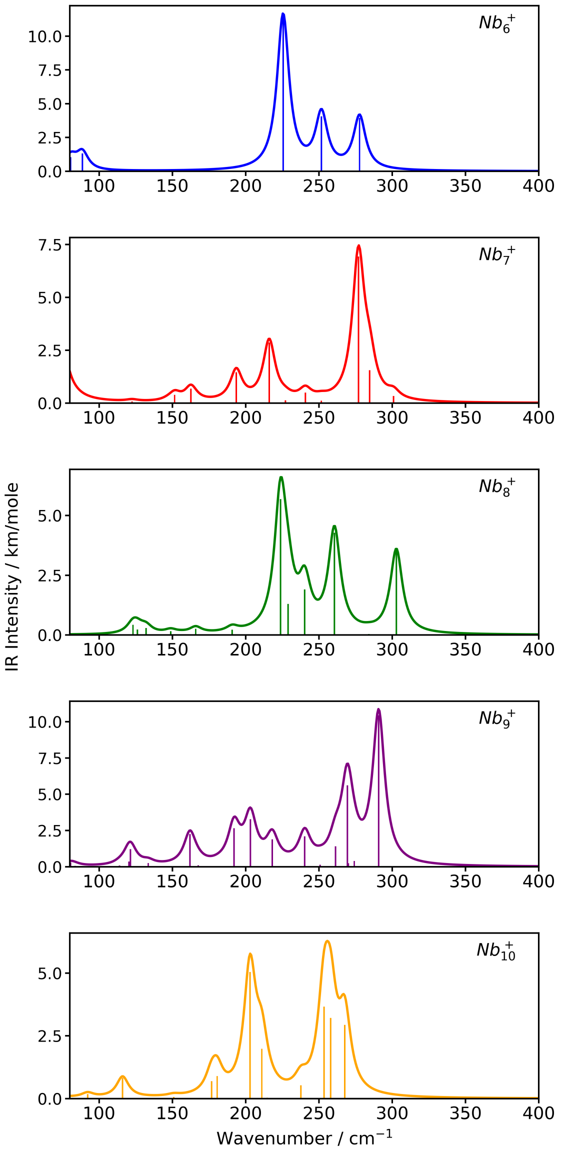

Figure S3 Computed spectra in the 85-400 cm^{-1} range for the proposed ground state structures of Nb_n^+ (n=6-10) clusters.

3 Influence of the Ar tag on computed spectra.

The experimental IR-MPD spectra relate specifically to the Ar-tagged species, but the computational models described in the main text do not have the Ar tag present. This is consistent with the expectation that the weakly-bound Ar atom should have a relatively minor impact on the spectra, but in this section we explicitly test that assumption. In Figure S4, we show the experimental spectra of the $AlNb_n^+$ clusters alongside the computed spectra of the most stable isomers, with and without the Ar tag present. Where present, the Ar tag is bound to one of the Nb atoms lying on the compressed axis, as this is found to be the energetically preferred position. In the majority of cases the impact of the Ar tag on both frequencies and intensities is minimal, and does not affect our assignments. The only marginal impact is an increase in the intensity of modes around 225 cm^{-1} which correspond to motion of the Nb_2 unit perpendicular to the plane of the other atoms. The enhancement in intensity is most prominent in the 220 cm^{-1} band of $AlNb_5^+$, such that it is similar to the 280 cm^{-1} band, affording a somewhat stronger match to the experimental data. There is also a slight increase in the 220 cm^{-1} peak in $AlNb_6^+$ and the 230 cm^{-1} peak in $AlNb_8^+$.

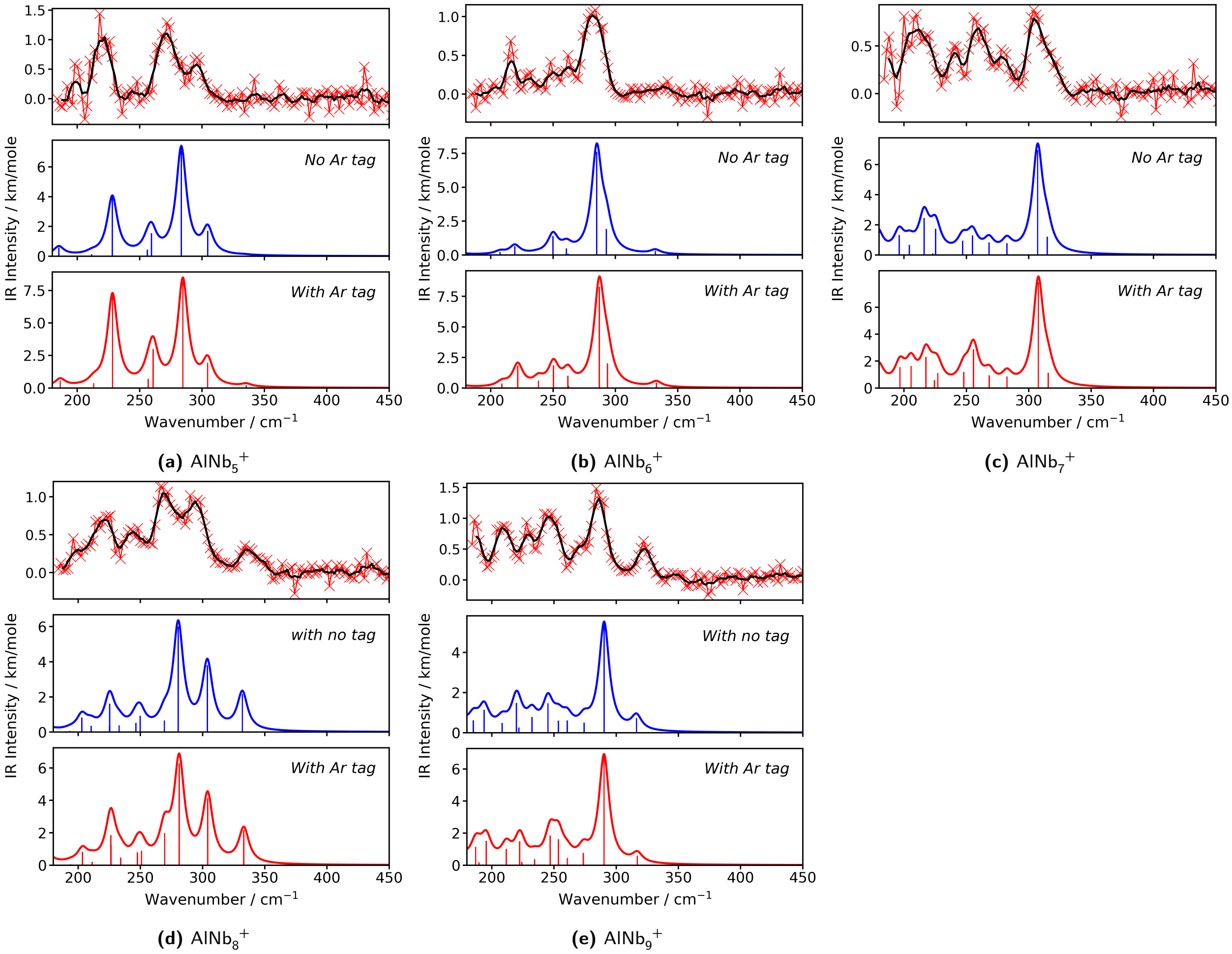

(a) $AlNb_5^+$ **(b)** $AlNb_6^+$ **(c)** $AlNb_7^+$

(d) $AlNb_8^+$ **(e)** $AlNb_9^+$

Figure S4 Comparison of spectra of $AlNb_n^+$ with (red) and without (blue) explicit inclusion of Ar tags in the computational model

4 Detailed Survey of the Potential Energy Landscape, including higher energy structures than those shown in the main text.

In the main text, only the lowest energy isomers were presented, but we have, in each case, carried out a systematic survey of the potential energy surface as set out in the experimental details. For clusters with an even number of electrons (those with 7, 9 or 11 atoms), we have considered singlets and triplets while for clusters with an odd number of electrons (6, 8 or 10 atoms) we have considered doublets and quartets. In the following figures, up to three additional isomers that lie within 1 eV of the equilibrioum structure are presented. Note that no additional isomers beyond those located in the main text were located for Nb_7^+, so there is no entry for that cluster in the Figure.

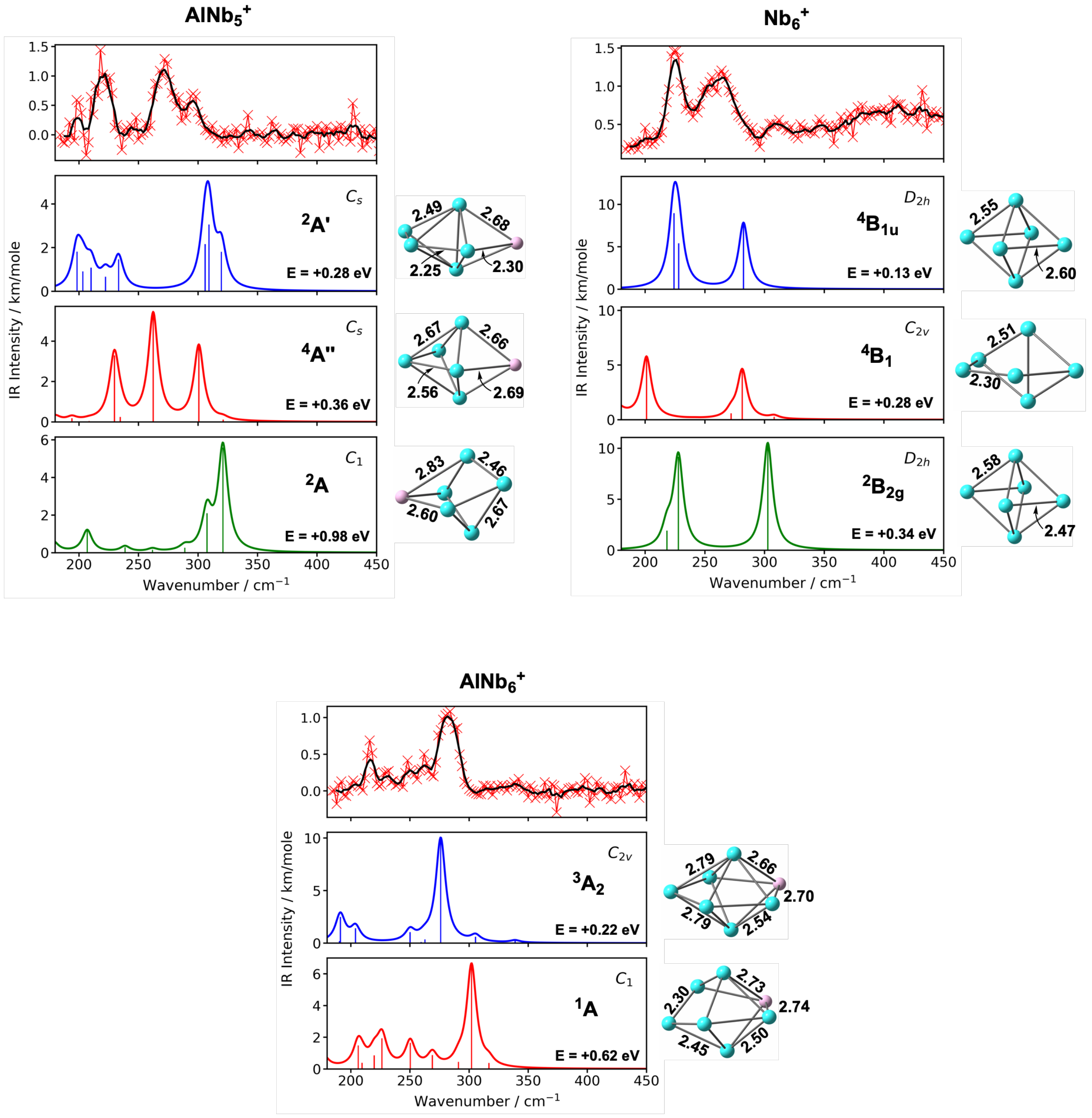

Figure S5 Measured IRMPD spectra of $AlNb_n^+ \cdot Ar$ and $Nb_n^+ \cdot Ar$ complexes and DFT computed vibrational spectra. The relative energies, spin state and structures are also presented.

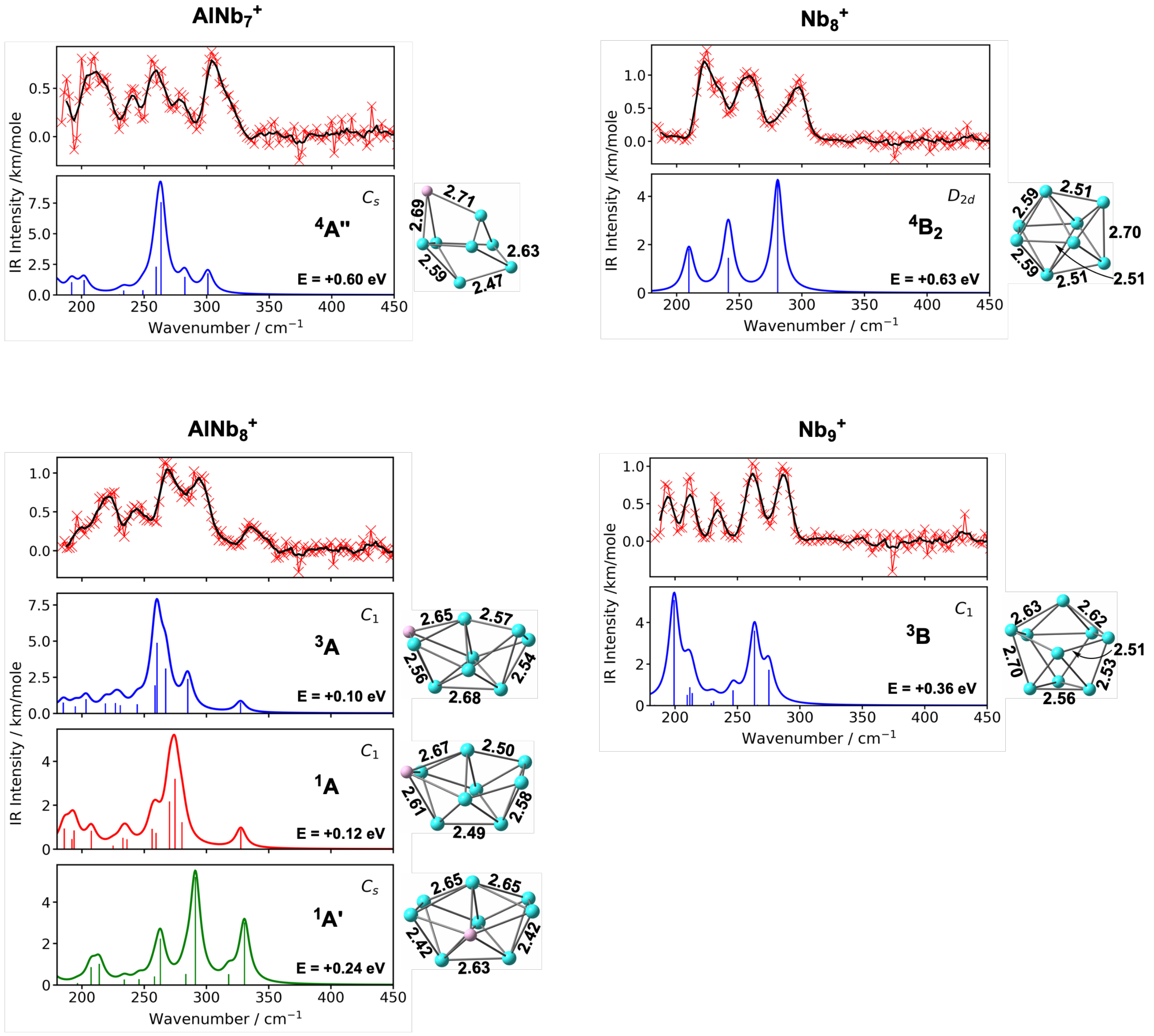

Figure S6 Measured IRMPD spectra of $AlNb_n^+ \cdot Ar$ and $Nb_n^+ \cdot Ar$ complexes and DFT computed vibrational spectra. The relative energies, spin state and structures are also presented.

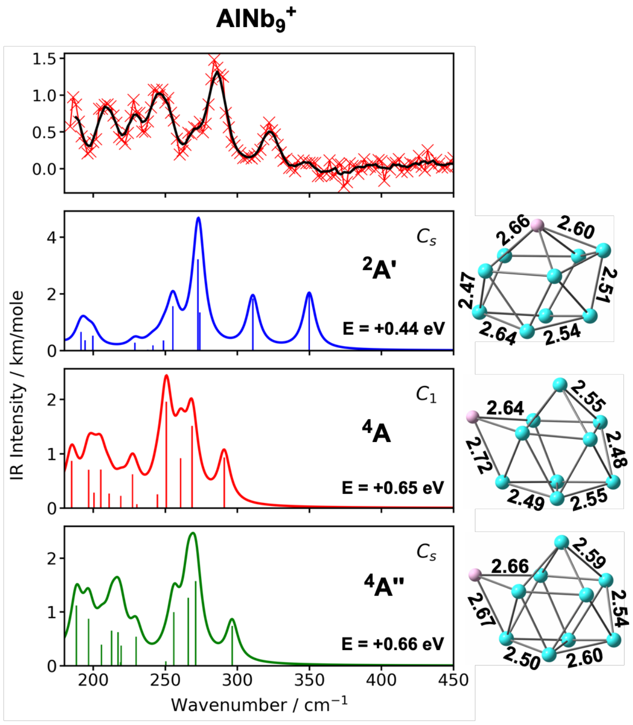

Figure S7 Measured IRMPD spectra of $AlNb_n^+ \cdot Ar$ and $Nb_n^+ \cdot Ar$ complexes and DFT computed vibrational spectra. The relative energies, spin state and structures are also presented.

5 Electronic Structure of the Nb_6^+ Cluster.

The axially compressed octahedron proves to be a common structural element in all the clusters from Nb_6^+ to Nb_8^+, and also in their Al-doped analogues, $AlNb_5^+$ to $AlNb_8^+$. The larger clusters can therefore be viewed as capped analogues of the Nb_6^+ and $AlNb_5^+$ base units, and the fingerprint of the *trans* Nb_2 unit can be tracked through the vibrational spectra of the entire family. Only at the 10-vertex level (Nb_{10}^+ and $AlNb_9^+$) does the capped, compressed octahedral motif give way to the more open square bi-capped antiprismatic structure, with larger central cavities. Given the apparent ubiquity of the axially compressed octahedron throughout this family, it is useful to explore its origins. A full account of the electronic structure of the metal clusters of different size, shape and composition will be the subject of a subsequent paper, but the key points as they relate to the vibrational spectroscopy can be illustrated most easily using the highly symmetric Nb_6^+ cluster, where we can follow a descent in symmetry from a perfect octahedron (O_h) to a D_{4h} symmetric structure that is compressed along one 4-fold axis and, finally, to the D_{2h}- and C_{2v}-symmetric structures that are almost iso-energetic (as we did for the vibrational spectra in Figure 2).

If the geometry of the Nb_6^+ cluster is constrained to perfect O_h symmetry, the valence-electron count of 29 generates a $^2T_{1u}$ ground state shown in Figure S8. The orbital degeneracy of the ground state immediately signals that a low-symmetry distortion will occur through the Jahn-Teller theorem, but the very general appearance of the axial compression across the whole family of clusters suggests that its origins are not linked specifically to the high degeneracy in an octahedron. Indeed, we find that the structure of Nb_6^{2+}, an orbital singlet, has a very similar axially-compressed structure. Each Nb atom delivers one 5*s* and five 4*d* orbitals, giving 36 valence orbitals in total, and we can divide the

4d orbitals into four distinct sets based on their local symmetry with respect to the vector connecting the atom to the centre of the cluster. The radially-directed d_{z^2} set has local σ symmetry and transforms as $a_{1g} \oplus e_g \oplus t_{1u}$, as do the linear combinations of 5s orbitals. The d_{xz} and d_{yz} orbitals have local π symmetry and transform as $t_{1g} \oplus t_{2g} \oplus t_{1u} \oplus t_{2u}$. The $d_{x^2-y^2}$ and d_{xy} orbitals both have local δ symmetry, but they are symmetry distinct because the former aligns along the Nb-Nb bonds whereas the latter are directed towards the centre of the Nb_3 faces. The $d_{x^2-y^2}$ set transform as $a_{2g} \oplus e_g \oplus t_{1u}$ while d_{xy} transform as $a_{2u} \oplus e_u \oplus t_{2g}$. The lowest energy orbitals in the manifold in Figure S8 are the completely in-phase combinations of 4d_{z^2} and 6s, $1a_{1g}$ and $2a_{1g}$, respectively. Also very low in energy is the $1a_{2u}$ orbital of δ symmetry localised on the Nb_3 faces. There then follows a band of eight orbitals, $1t_{1u}$, $1t_{2g}$ and $1e_g$, with Nb-Nb bonding character which are localised on the 12 edges of the octahedron. Collectively, these 11 orbitals accommodate 22 of the 29 valence electrons. 1 eV higher in energy lies a band of three triply-degenerate, approximately non-bonding, orbitals, $1t_{2u}$, $2t_{1u}$ and $2t_{2g}$, between which the remaining 7 valence electrons are distributed. It is this near-degeneracy, and the low-symmetry distortions that inevitably result from it, that is, ultimately the cause of the complex structural chemistry of the Nb_6 clusters.

The key orbitals that drive the axial compression of the octahedron in Nb_6^+ are $2t_{1u}$, an out-of-phase combination of radial d_{z^2} orbitals, and $1t_{2u}$ which, from our symmetry analysis above, can have $d_{x^2-y^2}$ (δ) and/or $d_{xz/yz}$ (π) character. The isosurface plot shows that the $1t_{2u}$ orbital has dominant δ character, with only a small degree of π character, while the opposite is true for the antibonding $2t_{2u}$ counterpart. The reason for the relatively clean separation between δ and π character is that, despite their common symmetry, the overlap between the two sets of orbitals is small: the $d_{xz/yz}$ orbitals on the equatorial atoms are not aligned optimally for overlap with $d_{x^2-y^2}$ on the axial atoms and *vice versa*. The axial compression splits the $1t_{2u}$ orbital into b_{2u} and e_u components, the first of which is strongly stabilised because the δ-π overlap is enhanced by shifting the axial Nb towards the equatorial plane (see the Walsh diagram in Figure S8). There are, of course, two other components of the $1t_{2u}$ orbital which correlate with e_u in D_{4h} symmetry, and these are destabilised for precisely the opposite reason: the axial compression reduces the π-δ overlap. The near-degenerate $2t_{1u}$ orbital is also split by the compression, into a_{2u} and e_u; the former is strongly destabilised as the Nb-Nb distance decreases. The opposite trajectories of the e_u components derived from $1t_{2u}$ and $2t_{1u}$ (shown as dashed lines in Figure S8) leads to extensive mixing, to the extent that, in the compressed structure, the occupied $2e_u$ orbital has dominant $2t_{1u}$ character, while $3e_u$ resembles the $1t_{1u}$ orbital. In effect, the $1b_{2u}{}^2 2e_u{}^4 3e_u{}^1$ configuration in the axially compressed form correlates with $1t_{2u}{}^3 2t_{1u}{}^4$ at the octahedral limit. The enhanced Nb_{ax}-Nb_{eq} bonding in the $1b_{2u}$ orbital leads to a contraction of the eight axial-equatorial Nb-Nb distances from 2.65 Å in the octahedron to 2.52 Å in the compressed geometry, and this leads indirectly to the contraction of the *trans*-annular Nb-Nb distance from 3.74 Å to 3.01 Å.

The very large difference in energy between the perfect octahedron and the compressed structure (1.78 eV) is clear evidence that the driving force is very strong, consistent with the fact that it persists into the larger clusters with 7, 8 and even 9 vertices, despite the perturbation by substitution of Nb for Al or the addition of capping atoms. It is striking that the Al substituent in the mixed $AlNb_n^+$ clusters ($n = 5,6,7,8$) always avoids the short *trans* sites - this is simply because the Al lacks d orbitals, and so the δ-π overlap that stabilises the $1b_{2u}$ orbital (or its equivalent in lower symmetry) is not possible when one of the Nb atoms is replaced by Al. An analysis of the topology of the electron density *via* the quantum theory of atoms in molecules (QTAIM) suggests that, despite the short Nb-Nb separation, there is no direct bond between these mutually *trans* Nb atoms and hence it is the contraction of the Nb_{ax}-Nb_{eq} bonds rather than the Nb_{ax}-Nb_{ax} distance

that stabilises the axially-compressed structure. Nevertheless, the short Nb_{ax}-Nb_{ax} distances effectively constrain the Nb_2 unit, such that it can be treated as a rigid rod in our interpretation of the vibrational spectra.

Even after the axial compression to generate the D_{4h}-symmetric structure, the ground state of $Nb_6{}^+$ remains orbitally degenerate (^2E_u), and therefore susceptible to a further 1st-order Jahn-Teller distortion. In the D_{2h}-symmetric form, this takes the form of a square-to-rectangle distortion in the equatorial plane, giving the $^2B_{3u}$ ground state shown in Figure 2 that lies only 0.07 eV below the D_{4h} isomer. In the main text, we have highlighted the debate in the literature over the identity of the ground state, and indeed we find, as do many other authors, that the C_{2v}-symmetric isomer, the 'dimer-capped rhombus', is marginally more stable than the D_{2h} form. However, when viewed from the perspective shown in Figure 2, it is clear that the two isomers share the short *trans*-annular Nb-Nb distance, and both can also be considered as a distorted variants of the same axially-compressed octahedron. In the C_{2v} isomer the distortion elongates a single edge rather than two parallel edges as in the D_{2h} isomer. The underlying driving force is, however, the same: both distortion modes lift the residual degeneracy of the e_u orbital, while avoiding any disruption of the short *trans*-annular separation. The relative energies of the different isomers of $Nb_6{}^+$ in Figure 2 show that the axial compression of the octahedron is far more significant than the subsequent distortion from D_{4h} to either D_{2h} or C_{2v}. In this way, we can view the two low-symmetry isomers as being descended from the same axially compressed octahedron, differing only in the subtle details of the final (and energetically rather insignificant) Jahn-Teller distortion of the ^2E_u state. It is perhaps unsurprising that their energies are similar, and that the precise order is sensitive to the details of the computation.

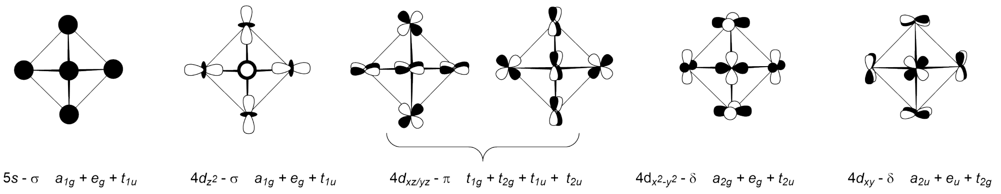

(a) Orbital symmetries.

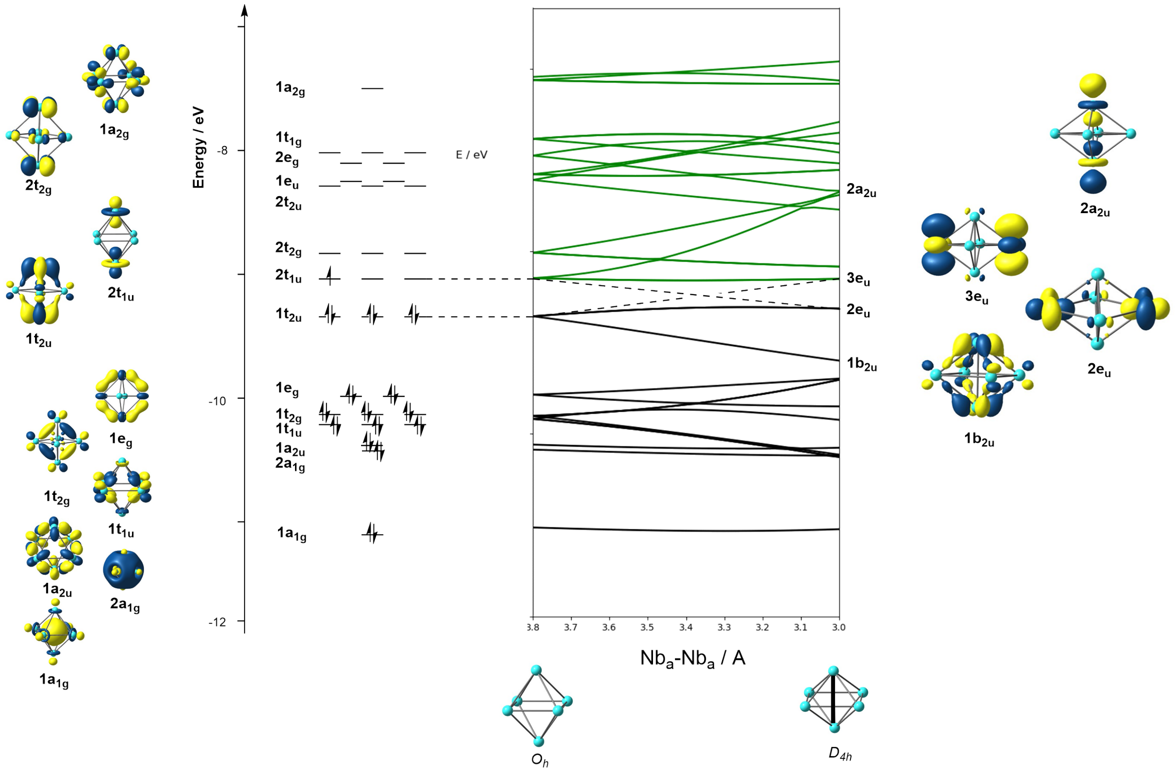

(b) Molecular orbital array.

Figure S8 (a) Orbital symmetries (b) and molecular orbital array for octahedral Nb_6^+, showing evolution in orbital energies as the symmetry is reduced from $O_h \rightarrow D_{4h}$.

6 Total energies and XYZ coordinates

Title: Nb_6^+ C_{2v}

Molecular Formula: Nb6

Charge: 1.0

Polarization (alpha-beta = MS): 1.0

functional: PBE

SCF Energy: -1.435586 au -39.0643 eV -900.84 kcal/mol -3769.13 kJ/mol

Optimized cartesian coordinates in Angstrom:

Nb	-0.0000	1.1625	1.4797
Nb	1.4021	0.0000	-0.7114
Nb	0.0000	2.0856	-0.7683
Nb	0.0000	-1.1625	1.4797
Nb	-1.4021	-0.0000	-0.7114
Nb	-0.0000	-2.0856	-0.7683

Title: Nb_6^+ D_{2h}

Molecular Formula: Nb6

Charge: 1.0

Polarization (alpha-beta = MS): 1.0

functional: PBE

SCF Energy: -1.432806 au -38.9886 eV -899.10 kcal/mol -3761.83 kJ/mol

Optimized cartesian coordinates in Angstrom:

Nb	1.3870	-1.4760	0.0000
Nb	1.3870	1.4760	0.0000
Nb	-0.0000	0.0000	1.5093
Nb	-1.3870	1.4760	0.0000
Nb	0.0000	0.0000	-1.5093
Nb	-1.3870	-1.4760	0.0000

Title: Nb_6^+ D_{4h}

Molecular Formula: Nb6

Charge: 1.0

Polarization (alpha-beta = MS): 1.0

functional: PBE

SCF Energy: -1.430081 au -38.9145 eV -897.39 kcal/mol -3754.68 kJ/mol

Optimized cartesian coordinates in Angstrom:

Nb	-1.4320	1.4320	0.0000
Nb	1.4320	1.4320	0.0000
Nb	0.0000	0.0000	1.5063
Nb	1.4320	-1.4320	0.0000
Nb	-0.0000	0.0000	-1.5063
Nb	-1.4320	-1.4320	0.0000

Title: Nb_6^+ Oh

Molecular Formula: Nb6

Charge: 1.0

Polarization (alpha-beta = MS): 1.0

functional: PBE

SCF Energy: -1.367340 au -37.2072 eV -858.02 kcal/mol -3589.95 kJ/mol

Optimized cartesian coordinates in Angstrom:

Nb	-1.8708	0.0000	0.0000
Nb	0.0000	-1.8708	0.0000
Nb	0.0000	0.0000	-1.8708
Nb	1.8708	0.0000	0.0000
Nb	0.0000	0.0000	1.8708
Nb	0.0000	1.8708	0.0000

Title: $AlNb_5^+$ C_{2v}

Molecular Formula: AlNb5

Charge: 1.0

Polarization (alpha-beta = MS): 1.0

functional: PBE

SCF Energy: -1.267727 au -34.4966 eV -795.51 kcal/mol -3328.42 kJ/mol

Optimized cartesian coordinates in Angstrom:

Nb	-0.0000	1.4949	0.2112
Nb	0.0000	0.0000	-1.8158
Nb	1.9426	0.0000	0.3502
Nb	0.0000	-1.4949	0.2112
Nb	-1.9426	-0.0000	0.3502
Al	0.0000	0.0000	2.3871

Title: $AlNb_5^+$ C_{4v}

Molecular Formula: AlNb5

Charge: 1.0

Polarization (alpha-beta = MS): 1.0

functional: PBE

SCF Energy: -1.229220 au -33.4488 eV -771.35 kcal/mol -3227.32 kJ/mol

Optimized cartesian coordinates in Angstrom:

Nb	0.0000	1.7601	0.2785
Nb	-0.0000	0.0000	-1.8010
Nb	1.7601	-0.0000	0.2785
Nb	-0.0000	-1.7601	0.2785
Nb	-1.7601	0.0000	0.2785
Al	-0.0000	0.0000	2.3810

Title: Nb_7^+ Cs ($^3A'$/)

Molecular Formula: Nb7

Charge: 1.0

Polarization (alpha-beta = MS): 2.0

functional: PBE

SCF Energy: -1.780085 au -48.4386 eV -1117.02 kcal/mol -4673.61 kJ/mol

Optimized cartesian coordinates in Angstrom:

Nb	-0.6971	-2.0091	-0.0000
Nb	0.1813	-0.7385	2.0036
Nb	0.1634	1.7326	1.2915
Nb	0.1635	1.7326	-1.2914
Nb	0.1813	-0.7385	-2.0036
Nb	1.5597	-0.3090	-0.0000
Nb	-1.5522	0.3298	-0.0000

Title: Nb_7^+ C_s ($^1A'$)

Molecular Formula: Nb7

Charge: 1.0

functional: PBE

SCF Energy: -1.777944 au -48.3803 eV -1115.68 kcal/mol -4667.99 kJ/mol

Optimized cartesian coordinates in Angstrom:

Nb	-2.0522	0.4228	0.0000
Nb	-0.8040	-0.2378	2.0136
Nb	1.6694	0.0196	1.4109
Nb	1.6694	0.0196	-1.4109
Nb	-0.8040	-0.2378	-2.0136
Nb	0.1110	-1.5557	0.0000
Nb	0.2105	1.5694	0.0000

Title: $AlNb_6^+$ C_2

Molecular Formula: AlNb6

Charge: 1.0

functional: PBE

SCF Energy: -1.614235 au -43.9256 eV -1012.95 kcal/mol -4238.17 kJ/mol

Optimized cartesian coordinates in Angstrom:

Nb	2.0344	-0.0342	0.7750
Nb	1.1350	0.2755	-1.5787
Nb	-1.1350	-0.2755	-1.5787
Nb	-2.0344	0.0342	0.7750
Nb	0.0081	-1.5104	0.4253
Nb	-0.0081	1.5104	0.4253
Al	0.0000	-0.0000	2.6057

Title: Nb_8^+ C_{2v}

Molecular Formula: Nb8

Charge: 1.0

Polarization (alpha-beta = MS): 1.0

functional: PBE

SCF Energy: -2.111073 au -57.4452 eV -1324.72 kcal/mol -5542.62 kJ/mol

Optimized cartesian coordinates in Angstrom:

Nb	1.4696	-0.0000	-1.8491
Nb	2.0113	-0.0000	0.6340
Nb	-0.0000	1.6311	-0.6171
Nb	-2.0113	0.0000	0.6340
Nb	0.0000	-1.6311	-0.6171
Nb	-1.4696	0.0000	-1.8491
Nb	0.0000	1.2455	1.8322
Nb	-0.0000	-1.2455	1.8322

Title: $Nb_8{}^+$ D_2d

Molecular Formula: Nb8

Charge: 1.0

Polarization (alpha-beta = MS): 1.0

functional: PBE

SCF Energy: -2.079967 au -56.5988 eV -1305.20 kcal/mol -5460.95 kJ/mol

Optimized cartesian coordinates in Angstrom:

Nb	0.9565	0.9565	1.8387
Nb	1.3012	1.3012	-0.6289
Nb	1.3012	-1.3012	0.6289
Nb	-1.3012	-1.3012	-0.6289
Nb	-1.3012	1.3012	0.6289
Nb	-0.9565	-0.9565	1.8387
Nb	0.9565	-0.9565	-1.8387
Nb	-0.9565	0.9565	-1.8387

Title: $AlNb_7{}^+$ C_s (1^2A_1)

Molecular Formula: AlNb7

Charge: 1.0

Polarization (alpha-beta = MS): 1.0

functional: PBE

SCF Energy: -1.923483 au -52.3406 eV -1207.00 kcal/mol -5050.10 kJ/mol

Optimized cartesian coordinates in Angstrom:

Nb	1.2305	-1.7344	0.0000
Nb	0.5940	0.1074	1.6217
Nb	-1.8136	0.7830	1.2114

Nb	-1.2443	-1.4293	0.0000
Nb	-1.8136	0.7830	-1.2114
Nb	0.5940	0.1074	-1.6217
Nb	2.4062	0.7275	0.0000
Al	0.1609	2.2569	0.0000

Title: $AlNb_7{}^+$ C_s (2^2A_1)

Molecular Formula: AlNb7

Charge: 1.0

Polarization (alpha-beta = MS): 1.0

functional: PBE

SCF Energy: -1.908949 au -51.9452 eV -1197.88 kcal/mol -5011.95 kJ/mol

Optimized cartesian coordinates in Angstrom:

Nb	-1.5580	0.6773	-1.2512
Nb	0.4226	1.8324	0.0000
Nb	2.3423	0.3790	0.0000
Nb	0.5409	-0.5586	-1.6022
Nb	0.5409	-0.5586	1.6022
Nb	-1.1826	-1.7742	0.0000
Nb	-1.5580	0.6773	1.2512
Al	1.5565	-2.3231	0.0000

Title: $Nb_9{}^+$ C_2

Molecular Formula: Nb9

Charge: 1.0

Polarization (alpha-beta = MS): 0.0

functional: PBE

SCF Energy: -2.427948 au -66.0678 eV -1523.56 kcal/mol -6374.58 kJ/mol

Optimized cartesian coordinates in Angstrom:

Nb	-0.0072	2.1918	-0.2452
Nb	0.0000	0.0000	1.5958
Nb	0.0072	-2.1918	-0.2452
Nb	-1.2033	-0.0227	-1.5839
Nb	1.2033	0.0227	-1.5839
Nb	2.2267	1.1500	0.3664
Nb	2.0359	-1.3407	0.6648
Nb	-2.2267	-1.1500	0.3664
Nb	-2.0359	1.3407	0.6648

Title: $AlNb_8^+$ C_1 (1^1A)

Molecular Formula: AlNb8

Charge: 1.0

Polarization (alpha-beta: = MS): 0.0

functional: PBE

SCF Energy: -2.237093 au -60.8744 eV -1403.80 kcal/mol -5873.49 kJ/mol

Optimized cartesian coordinates in Angstrom:

Nb	-0.2230	2.1740	0.7810
Nb	-1.7969	-0.2165	0.9303
Nb	-0.1452	-2.1184	0.7681
Nb	0.0046	0.4330	2.5337
Nb	1.4420	-0.1637	0.6322
Nb	2.0492	-1.5094	-1.6289
Nb	-1.6881	1.2354	-1.0950
Nb	-0.5489	-1.0489	-1.4807
Nb	0.9062	1.2146	-1.4407

Title: $AlNb_8^+$ C_1 (2^1A)

Molecular Formula: AlNb8

Charge: 1.0

Polarization (alpha-beta: = MS): 0.0

functional: PBE

SCF Energy: -2.234757 au -60.8108 eV -1402.33 kcal/mol -5867.35? kJ/mol

Optimized cartesian coordinates in Angstrom:

Nb	-0.0335	2.0308	-0.5015
Nb	0.1356	0.1692	1.2774
Nb	-0.1864	-2.0606	-0.6272
Nb	-1.3109	-0.0740	-1.5175
Nb	1.4670	0.0669	-1.3653
Nb	2.5102	1.2514	0.7415
Nb	2.0096	-1.4071	0.5639
Nb	-2.1703	-1.1256	0.9148
Nb	-2.4212	1.1490	0.5138

Title: $Nb_{10}{}^{+}$ D_2

Molecular Formula: Nb10

Charge: 1.0

Polarization (alpha-beta = MS): 1.0

functional: PBE

SCF Energy: -2.761025 au -75.1313 eV -1732.57 kcal/mol -7249.07 kJ/mol

Optimized cartesian coordinates in Angstrom:

Nb	0.0000	-0.0000	-2.6091
Nb	-0.0000	-0.0000	2.6091
Nb	-1.8189	-0.9329	-0.9896
Nb	1.8189	0.9329	-0.9896
Nb	-0.8770	1.7527	-1.0665
Nb	0.8770	-1.7527	-1.0665
Nb	-0.8770	-1.7527	1.0665

Nb	0.8770	1.7527	1.0665
Nb	-1.8189	0.9329	0.9896
Nb	1.8189	-0.9329	0.9896

Title: Nb_{10}^{+} D_4

Molecular Formula: Nb10

Charge: 1.0

Polarization (alpha-beta = MS): 1.0

functional: PBE

SCF Energy: -2.760262 au -75.1105 eV -1732.09 kcal/mol -7247.07 kJ/mol

Optimized cartesian coordinates in Angstrom:

Nb	0.0000	0.0000	-2.5563
Nb	-0.0000	-0.0000	2.5563
Nb	-1.8287	-0.8395	-1.0178
Nb	1.8287	0.8395	-1.0178
Nb	-0.8395	1.8287	-1.0178
Nb	0.8395	-1.8287	-1.0178
Nb	-0.8395	-1.8287	1.0178
Nb	0.8395	1.8287	1.0178
Nb	-1.8287	0.8395	1.0178
Nb	1.8287	-0.8395	1.0178

Title: $AlNb_9^{+}$ C_1 (^2A)

Molecular Formula: AlNb9

Charge: 1.0

Polarization (alpha-beta = MS): 1.0

functional: PBE

SCF Energy: -2.557515 au -69.5935 eV -1604.87 kcal/mol -6714.76 kJ/mol

Optimized cartesian coordinates in Angstrom:

Nb	-0.1225	1.3473	-0.0645
Nb	-1.3395	0.3624	-2.0792
Nb	1.2018	0.9084	2.0716
Nb	-1.3200	0.1899	2.1627
Nb	1.4404	0.6674	-2.1299
Nb	0.3312	-1.4826	-1.3270
Nb	0.4049	-1.5117	1.2376
Nb	-2.0439	-0.9257	0.0198
Nb	2.3604	0.0430	0.0410
Al	-2.8055	1.5918	0.2416

Title: $AlNb_9^+$ C_s ($^2A'$)

Molecular Formula: AlNb9

Charge: 1.0

Polarization (alpha-beta = MS): 1.0

functional: PBE

SCF Energy: -2.548810 au -69.3566 eV -1599.40 kcal/mol -6691.90 kJ/mol

Optimized cartesian coordinates in Angstrom:

Nb	2.8292	0.4033	0.0000
Nb	-2.5804	-0.4082	0.0000
Nb	1.2748	-1.7946	0.0000
Nb	1.1411	0.3755	1.7547
Nb	1.1411	0.3755	-1.7547
Nb	-1.3124	1.5089	-1.2185
Nb	-0.7294	-1.3333	1.4301
Nb	-1.3124	1.5089	1.2185
Nb	-0.7294	-1.3333	-1.4301
Al	0.9562	2.4009	0.0000

7 Sample ADF input file

A sample ADF input file (for $[AlNb_5]^+$) is shown below.

```
Task      GeometryOptimization
System
ATOMS
  Al    0.000000000    0.000000000    2.387461386
  Nb    0.000000000    1.496134746    0.211228057
  Nb    0.000000000    0.000000000   -1.814717423
  Nb    1.943747408    0.000000000    0.349451631
  Nb    0.000000000   -1.496134746    0.211228057
  Nb   -1.943747408    0.000000000    0.349451631
END
Charge      1
Symmetrize      Yes
SYMMETRY      C(s)
End

Usesymmetry      Yes

Properties
  NormalModes      Yes
End
NormalModes
  Hessian      Analytical
end

Engine      ADF
  basis
      type      TZ2P
      FitType      QZ4P
      core      None
      createoutput      No
  End
  BECKEGRID
      Quality      Good   End
  Unrestricted      Yes
  SpinPolarisation      1.0
  XC
      GGA      PBE
  End
  Analyticalfreq
      max_cpks_iterations      100
  End
  Relativity
  Formalism      ZORA
  End
Endengine
```

Notes and references

[1] A. Fielicke, C. Ratsch, G. von Helden and G. Meijer, *J. Chem. Phys.*, 2007, **127**, 234306.
